# Using coligands to gain mechanistic insight into iridium complexes hyperpolarized with *para*-hydrogen†
†Electronic supplementary information (ESI) available. CCDC 1893624. For ESI and crystallographic data in CIF or other electronic format see DOI: 10.1039/c9sc00444k; NMR can be located *via*https://doi.org/10.15124/bbafb6fb-d40a-45ae-81c2-9bebe43249b2


**DOI:** 10.1039/c9sc00444k

**Published:** 2019-03-19

**Authors:** Ben. J. Tickner, Richard O. John, Soumya S. Roy, Sam J. Hart, Adrian C. Whitwood, Simon B. Duckett

**Affiliations:** a Center for Hyperpolarization in Magnetic Resonance (CHyM) , University of York , Heslington , York , YO10 5NY , UK . Email: simon.duckett@york.ac.uk; b Department of Chemistry , University of York , Heslington , York , YO10 5DD , UK

## Abstract

We report the formation of a series of novel [Ir(H)_2_(IMes)(α-^13^C_2_-carboxyimine)L] complexes in which the identity of the coligand L is varied. When examined with *para*-hydrogen, complexes in which L is benzylamine or phenethylamine show significant ^1^H hydride and ^13^C_2_ imine enhancements and may exist in ^13^C_2_ singlet spin order. Isotopic labeling techniques are used to double ^13^C_2_ enhancements (up to 750-fold) and singlet state lifetimes (up to 20 seconds) compared to those previously reported. Exchange spectroscopy and Density Functional Theory are used to investigate the stability and mechanism of rapid hydrogen exchange in these complexes, a process driven by dissociative coligand loss to form a key five coordinate intermediate. When L is pyridine or imidazole, competitive binding to such intermediates leads to novel complexes whose formation, kinetics, behaviour, structure, and hyperpolarization is investigated. The ratio of the observed PHIP enhancements were found to be affected not only by the hydrogen exchange rates but the identity of the coligands. This ligand reactivity is accompanied by decoherence of any ^13^C_2_ singlet order which can be preserved by isotopic labeling. Addition of a thiol coligand proved to yield a thiol oxidative addition product which is characterized by NMR and MS techniques. Significant 870-fold ^13^C enhancements of pyridine can be achieved using the Signal Amplification By Reversible Exchange (SABRE) process when α-carboxyimines are used to block active coordination sites. [Ir(H)_2_(IMes)(α-^13^C_2_-carboxyimine)L] therefore acts as unique sensors whose ^1^H hydride chemical shifts and corresponding hyperpolarization levels are indicative of the identity of a coligand and its binding strength.

## Abstract 2

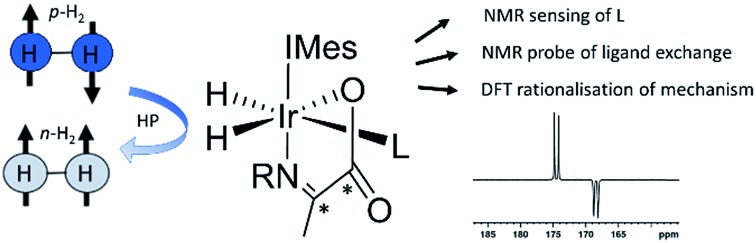
Hyperpolarised iridium carboxyimine complexes yield diagnostic signals whilst undergoing ligand exchange processes rationalised by NMR and DFT.

## Introduction

Nuclear Magnetic Resonance (NMR) is one of the most widely used techniques in the structural and behavioural characterization of molecules and materials. Despite its widespread use it remains fundamentally insensitive as its signal intensity is derived from very small population differences across closely spaced nuclear spin energy levels. While only 1 in 32 000 ^1^H nuclei are visible to NMR at 9.4 T, this problem becomes more pronounced for low γ nuclei such as ^13^C as only 1 in 800 000 nuclear spins contribute efficiently to the NMR response at the 1.5 T field used by a common clinical scanner. Hyperpolarization techniques have developed over the last few decades to address this insensitivity issue by premagnetising samples and hence creating non-Boltzmann population differences across their NMR addressable energy levels.^1^–^7^ Techniques such as Dynamic Nuclear Polarization (DNP),^1^,^2^ *para*-Hydrogen Induced Polarization (PHIP)^3^–^6^ and Spin Exchange Optical Pumping (SEOP)^7^ are used in this regard to produce hyperpolarized molecules with growing success.

The technique of DNP has already found applications in a clinical context as it can deliver ^1^H and ^13^C nuclei with polarization levels of 92% and 70% respectively after preparation times of 150 seconds and 20 minutes respectively.^2^ It exploits the transfer of polarization from an electron in a stable radical at cryogenic temperatures of between 1 and 2 K that is located in a magnetic field of between 1 and 7 T to achieve this.^1^,^2^ The successful hyperpolarization of biochemically relevant agents such as pyruvate,^8^–^14^ succinate,^15^,^16^ and fumarate^17^,^18^ and their subsequent *in vivo* detection when thawed reflects a significant advance in diagnostic magnetic resonance imaging (MRI). In addition, SEOP delivers hyperpolarized samples of the gases ^3^He and ^129^Xe which have been used for human lung imaging.^19^,^20^

The PHIP technique utilizes easy to access *p*-H_2_, the singlet nuclear spin isomer of hydrogen, to achieve hyperpolarization. This is readily realized when it is incorporated into a molecule by a hydrogenation reaction and has enabled the *in vivo* detection of MRI sensitised reaction products.^15^,^21^–^23^ Recently, a variant of PHIP, termed PHIP-SAH, employed the hydrogenation of a readily cleaved and unsaturated side arm that is attached to pyruvate or acetate to ultimately hyperpolarize them.^24^,^25^

In contrast, the PHIP method, Signal Amplification By Reversible Exchange (SABRE) rapidly hyperpolarizes substrates in a cost-efficient and reproducible fashion without the chemical alteration of the substrate.^26^–^28^ Substrate polarization is now facilitated by the temporary association of the target agent within an organometallic complex at low (0–100 G) magnetic field, although polarization transfer can be driven at high field by radio frequency excitation.^28^–^31^ The most commonly used substrates for SABRE contain N-heterocyclic motifs such as those found in pyridines,^26^,^32^–^34^ nicotinamides,^26^,^35^,^36^ and pyrazines.^32^,^33^ The metal binding restriction has been lifted by the SABRE-Relay approach which involves the chemical exchange of hyperpolarized nuclei.^37^–^39^

In fact, SABRE has achieved 63% ^1^H polarization in methyl-4,6-*d*_2_-nicotinate in just a few seconds and can therefore deliver a similar output to DNP.^40^ A SABRE-SHEATH variation, demonstrated for N-heterocycles,^35^,^41^ nitriles,^41^ Schiff bases,^42^ and diazirines,^43^,^44^ has targeted ^15^N nuclei through transfer in milli-Gauss fields. SABRE has also hyperpolarized ^13^C,^45^,^46^ ^19^F,^47^,^48^ ^31^P,^49^^119^Sn and ^31^Si^50^ nuclei and is therefore truly multinuclear in scope. Hence, PHIP and SABRE now find uses in a wide range of situations including reaction monitoring and the detection of low concentration analytes in mixtures or short lived intermediates in the field of catalysis.^3^,^4^ There are also an array of high sensitivity analytical applications^51^,^52^ alongside reports to produce biocompatible mixtures suitable for *in vivo* injection.^53^–^55^

**Chart 1:**
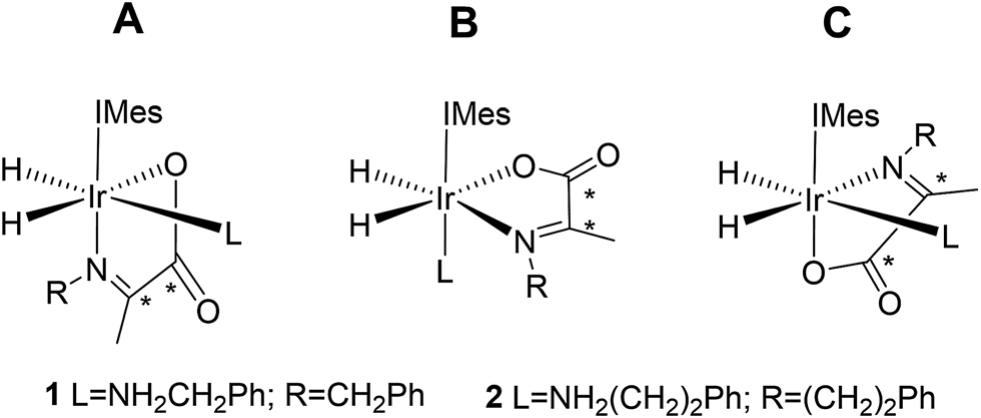
Structural isomers of the iridium α-carboxyimine complexes seen in this work. An asterisk (*) denotes a ^13^C labeled position. IMes = 1,3-bis(2,4,6-trimethyl-phenyl)imidazole-2-ylidene.

Normally in NMR, relaxation can be counted as a friend because it allows the user to signal average. However, for hyperpolarization relaxation is widely thought of as a foe because the non-equilibrium state it utilises must be encoded for measurement before it vanishes. Consequently, a number of methods have been developed to extend the detectible lifetimes of hyperpolarized signals through deuterium labeling^26^,^40^,^56^,^57^ and/or storage as singlet spin order.^58^–^60^ We communicated previously the formation and behaviour of two labelled iridium α-carboxyimine complexes that are hyperpolarized by PHIP and exist initially as ^13^C_2_ nuclear singlet states.^37^ These products result from binding of the imine formed by the *in situ* condensation reaction of amine and pyruvate. In solution, these [Ir(H)_2_(IMes)(η^2^-α-carboxyimine)(amine)] complexes (where IMes = 1,3-bis(2,4,6-trimethyl-phenyl)imidazole-2-ylidene) exist as two isomers, denoted **A** or **B** of Chart 1, that are differentiated according to the coordination geometry of the imine. Isomer, **C**, was not detected in this study.

Only those isomers denoted as **A** exhibited rapid hydrogen exchange and hence produced high levels of hydride and ^13^C_2_ signal enhancements under PHIP conditions. When comparing these complexes, **1A** was reported to undergo a faster rate of H_2_ loss (15.5 ± 0.6 s^–1^) when compared to **2A** (4.11 ± 0.06 s^–1^) at 283 K. This was suggested to account for the higher ^13^C signal enhancements it exhibits (420-fold for **1A***versus* 280-fold for **2A**). Its ^13^C_2_ singlet state also exhibited an increased lifetime (10.9 ± 1.1 s in **1A** compared to 8.8 ± 1.4 s for **2A**). Given the potential importance of hyperpolarised NMR for the rationalisation of reaction mechanisms and the characterisation of materials in low concentration, alongside their use in MRI as clinical diagnostics, we set out here to study these complexes in more detail. Specifically, we expected that these hyperpolarization levels and their visible lifetimes could be improved through optimization of the hydrogen loss rate and selective deuteration.^40^ We explore the hydrogen exchange pathway of these iridium carboxyimine complexes by studying them as a function of amine ligand concentration and hydrogen pressure through NMR spectroscopy methods. Results are then linked to a mechanism which is supported by Density Functional Theory (DFT) calculations. By studying the reactivity of these complexes towards the eight ligands of Chart 1 we form a series of novel complexes that allow us to further rationalise this behaviour. Subsequently their hyperpolarisation with *p*-H_2_ is examined and we establish a route to produce strong ^13^C signal gains. By expanding this work to include the effects of ^15^N and ^2^H isotopic labelling we develop further insight into the important SABRE mechanism.

## Results and discussion

This work starts with the formation of neutral [Ir(H)_2_(IMes)(η^2^-α-carboxyimine)(amine)] (**1** and **2**) which exist as two regioisomers that are differentiated according to whether the amine is *trans* to hydride (**A**) or the N-heterocyclic carbene ligand (**B**) as shown in Chart 1. These complexes are prepared *in situ* by taking dichloromethane-*d*_2_ solutions of a [Ir(IMes)(COD)Cl] precursor and reacting it with 5 equivalents of both pyruvate and the amine (see Experimental) in the presence of a 3 bar H_2_ atmosphere.^37^ The initial products of this process are iridium(iii) dihydride complexes of the type [Ir(H)_2_(IMes)(amine)_3_]Cl which have been reported to undergo both H_2_ loss and amine loss *via* the formation of a common 16 electron intermediate [Ir(H)_2_(IMes)(amine)_2_]Cl. These species go on to form **1** and **2** in the presence of pyruvate.

### Hydrogen loss mechanism for the iridium dihydride α carboxyimine complex **2A**

Upon the selective radio frequency (r.f.) excitation of a hydride resonance of the related phenylethylamine product **2A**, exchange of this hydride into both free H_2_ and the inequivalent hydride site is observed. This suggests the presence of a reaction pathway that allows for both H_2_ exchange and interchange of the hydride ligand sites. Modelling this exchange spectroscopy (EXSY) data allowed the experimentally observed rates of hydrogen production (*k*_(obs)H_2__) and hydride site interchange (*k*_(obs)Hi_) to be determined from the corresponding signal integrals as a function of reaction time. The results of this process are shown in Tables 1 and 2.

**Table 1:** Rate constants for H_2_ production (*k*_(obs)H_2__) and hydride interchange (*k*_(obs)Hi_) of **2A** determined by EXSY at 273 K as a function of amine concentration^*a*^ when H_2_ pressure was fixed at 3 bar

[Amine]	*k*_(obs)H_2__/s^–1^	*k*_(obs)Hi_/s^–1^
5 eq.	1.85 ± 0.05	0.40 ± 0.02
10 eq.	1.47 ± 0.03	0.70 ± 0.01
15 eq.	1.13 ± 0.01	0.88 ± 0.01

^*a*^Amine concentration is relative to the iridium precatalyst.

**Table 2:** Rate constants for H_2_ production (*k*_(obs)H_2__) and hydride interchange (*k*_(obs)Hi_) of **2A** determined by EXSY at 273 K as a function of H_2_ pressure when amine concentration was fixed at 15 eq. relative to iridium precatalyst

H_2_ pressure	*k*_(obs)H_2__/s^–1^	*k*_(obs)Hi_/s^–1^
1 bar	0.60 ± 0.01	1.05 ± 0.01
2 bar	0.95 ± 0.01	0.92 ± 0.01
3 bar	1.13 ± 0.01	0.88 ± 0.01

**Fig. 1:**
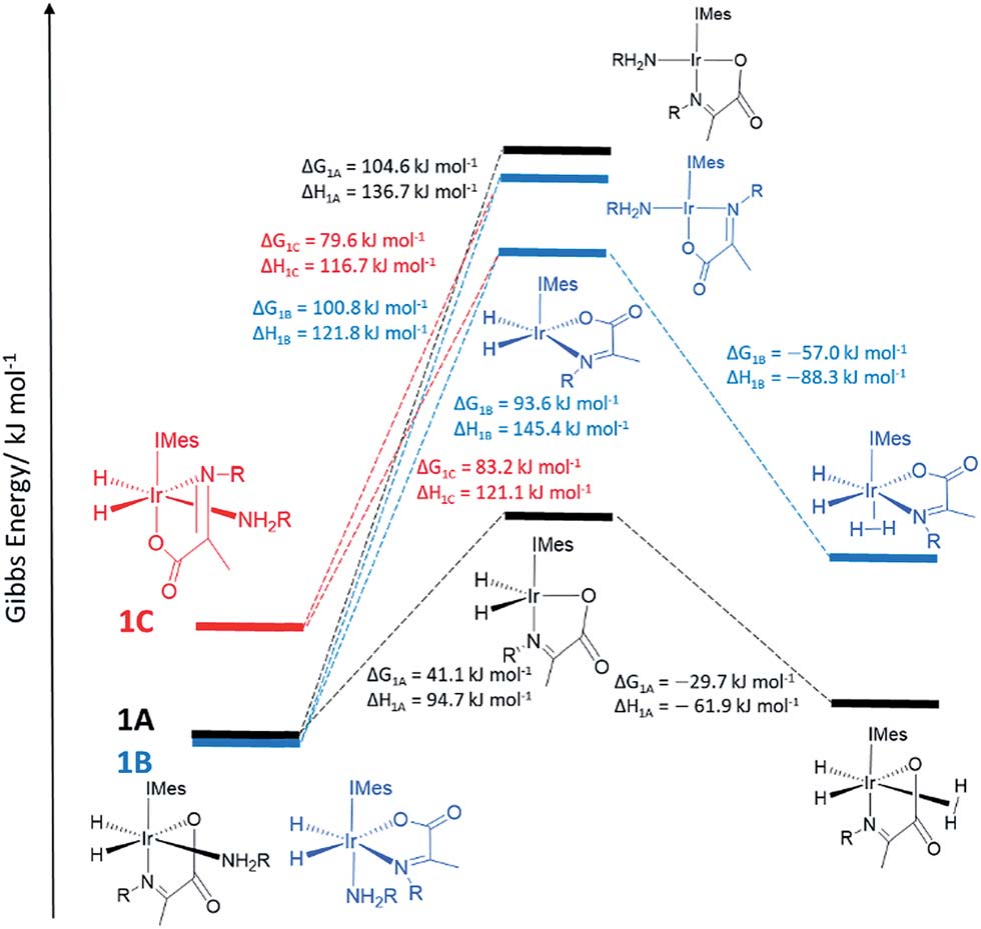
DFT energy level diagram supporting a hydrogen exchange pathway that is preceded by dissociative amine loss to form the indicated five coordinate intermediates. These are intermediate energies and do not reflect transition state barriers.

On the basis of similar Ir(iii) reactions, it might be expected that hydrogen exchange is again preceded by dissociative amine loss from 18 electron **2A** to form a 16 electron five coordinate intermediate.^33^ The *k*_(obs)H_2__ rate constants of Table 1 fall as the amine concentration increases. This change is consistent with the fact that the five coordinate intermediate [Ir(H)_2_(IMes)(η^2^-α-carboxyimine)] of Fig. 1 is more likely to reform **2A** at higher amine concentrations than react with H_2_ thereby reducing *k*_(obs)H_2__ and consequently increasing the value of *k*_(obs)Hi_. Subsequent rebinding of the amine reforms the starting complex **2A**, with or without hydride ligand interchange depending on the face of amine attack on the 16 electron intermediate. This subtle effect is a consequence of the fact the hydride ligands of the 16-electron intermediate are made chemically inequivalent by virtue of the imine asymmetry. In contrast, when the H_2_ pressure is increased the five coordinate intermediate is more likely to react with H_2_ than amine leading to more efficient H_2_ loss *via* [Ir(H)_2_(η^2^-H_2_)(IMes)(η^2^-α-carboxyimine)(amine)] which is reflected in an increase in *k*_(obs)H_2__ and a decrease in *k*_(obs)Hi_.

In order to provide further evidence for these deductions, Density Functional Theory (DFT) calculations were used to predict the relative energies of closely related **1A–1C** as detailed in Fig. 1. Their relative stability is **1A** ≥ **1B** ≫ **1C** which matches the solution based NMR observations. The energy changes associated with direct H_2_ loss to form the corresponding four coordinate Ir(i) 16-electron product are also shown in Fig. 1. The enthalpy and free energy changes of direct H_2_ loss are both in excess of 100 kJ mol^–1^ at 298 K making such a process energetically unfavourable. In contrast, amine loss to form the corresponding trigonal bipyramidal 16-electron Ir(iii) intermediate of Fig. 1 predicted earlier is more favourable by over 60 kJ mol^–1^ there-by supporting a route to H_2_ loss in a constant iridium oxidation state cycle involving [Ir(H)_2_(H_2_)(IMes)(η^2^-α-carboxyimine)] as shown in Fig. 1. The hydride ligands in the optimized geometry of these intermediates are inequivalent in agreement with the observed kinetic effects described earlier (Fig. S8 of ESI†). Additionally, the energy changes for amine loss are larger for isomer **B** in accordance with its experimentally observed reduced reactivity.

### Formation of analogous iridium α-carboxyimine complexes **3–5** from **2** by reaction with pyridine, imidazole and dimethylsulfoxide

**Scheme 1:**
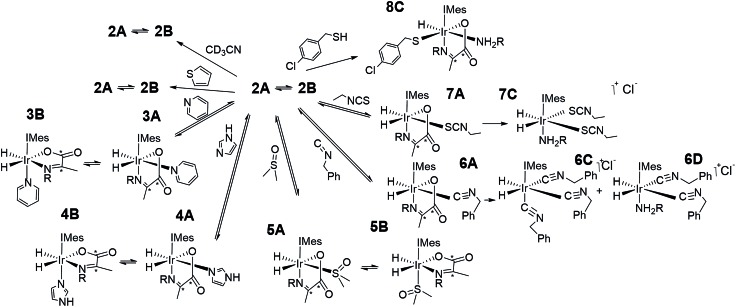
Complexes synthesised in this work.

As amine loss from **2A** mediates *p*-H_2_ exchange we mixed solutions of it with several neutral two electron donors to probe this process. These materials, hence forth referred to as co-ligands, are illustrated in Scheme 1 alongside the corresponding reaction products. It might reasonably be expected that trapping of the resulting 16-electron intermediate [Ir(H)_2_(IMes)(η^2^-α-carboxyimine)] would result in a number of new PHIP enhanced reaction products which could themselves have implications for SABRE.

Upon adding an ∼5-fold excess relative to iridium of one of the weak donors acetonitrile-*d*_3_ or thiophene to a sample of **2** no changes in the corresponding ^1^H NMR spectra are observed. Hence the predicted ligand exchange products must be unstable with respect to **2** and consequently the Ir–amine bond energy must exceed that of both Ir–acetonitrile and Ir–thiophene. This agrees with the corresponding DFT calculations (Fig. S8 of ESI†) which suggest these substitution products are at least 20 kJ mol^–1^ less stable than **2**.

**Fig. 2:**
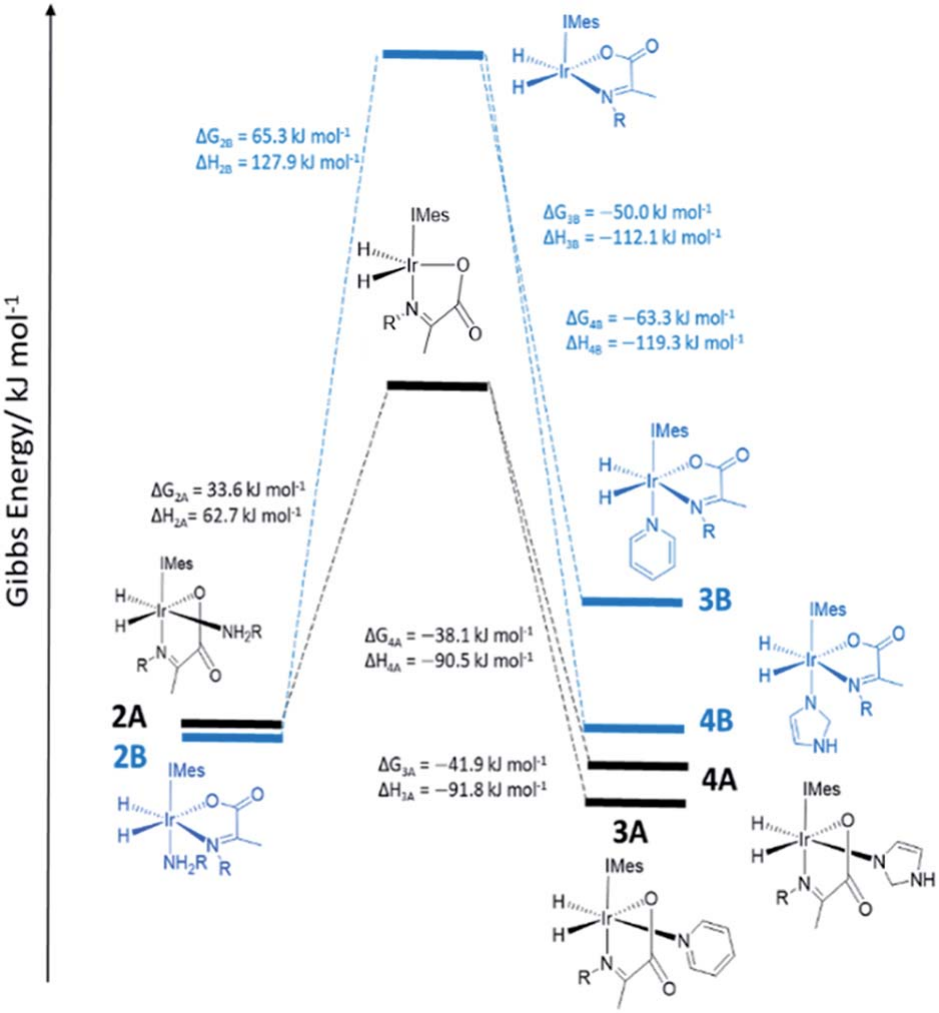
DFT energy level diagram for amine loss from **2** and subsequent binding of pyridine or imidazole coligands to the five coordinate intermediates indicated. Energies do not reflect transition state barriers.

However, when a 4-fold excess of pyridine is added to **2** in DCM-*d*_2_ two new hydride resonances immediately appear at *δ* –23.47 and *δ* –27.37 in the corresponding ^1^H NMR spectrum. These resonances exhibit a mutual *J*_HH_ coupling of 7.5 Hz and are therefore due to a *cis*-dihydride complex, the amine replacement product **3A**. Upon leaving this solution overnight at 278 K two further hydride resonances appear at *δ* –20.96 and *δ* –26.99, this time sharing a mutual coupling of 9 Hz, due to isomer **3B** of Scheme 1. Hence pyridine and phenylethylamine bind competitively to [Ir(H)_2_(IMes)(η^2^-α-carboxyimine)]. At this point in the reaction, hydride ligand ^1^H NMR signals are visible for all four of the associated complexes. A fresh sample was prepared to track these speciation changes at 298 K over a 17 hour time period by ^1^H NMR spectroscopy. Examination of these data revealed that the signals for **2A**, and then **2B**, decrease upon pyridine addition leading first to the detection of **3A** and then **3B**. The signals for **3B** are most readily seen in the later stages of reaction due to its low proportion in the final equilibrium mixture of all four species. These observations fit to a kinetic model (see ESI†) from which pseudo rate constants for their relative rates of transmission can be determined. The model does not involve the common 16-electron intermediate that forms regardless of the identity of the ligand that is lost. Collectively, these observations alongside the DFT predictions of Fig. 2 are consistent with faster amine loss from **2A** (transmission rate *k*_trans2A3A_ of 9.2 ± 1.8 × 10^–6^ s^–1^) than from **2B** (*k*_trans2B3B_ is 7.1 ± 2.9 × 10^–6^ s^–1^). According to the kinetic model *k*_trans2A3A_ is always greater than *k*_trans2B3B_. While these rates of transmission are similar, **3A** dominates over **3B** at equilibrium because it is more stable to pyridine loss (supported by DFT as shown in Fig. 2). The ratio of **3A** to **2** is, however, influenced by the associated equilibria which are complicated by the *in situ* pyruvate reaction to form the imine, alongside the fact DFT predicts them all to be close in energy.

Similar changes in the hydride region of analogous NMR spectra were seen when a solution of **2** is mixed with imidazole rather than pyridine. This is reflected in the appearance of two new mutually coupled hydride resonances at *δ* –21.96 and *δ* –28.14 (*J*_HH_ = 8 Hz) due to imidazole containing **4A** of Scheme 1. Isomer **4B** yields resonances at *δ* –21.41 and *δ* –27.59 (*J* = 9 Hz) and again becomes visible most readily at longer reaction times. Fitted transmission rate constants suggest again that there is a faster effective rate of amine replacement from **2A** to form **4A** (*k*_trans2A4A_ = 3.1 ± 0.4 × 10^–6^ s^–1^) when compared to **2B** (*k*_trans2B4B_ = 1.9 ± 0.4 × 10^–6^ s^–1^), although these changes proceed slower than those for pyridine. Hence the rate of imidazole binding to [Ir(H)_2_(IMes)(η^2^-α-carboxyimine)] must be slower than pyridine even though **4B** ultimately exists in higher proportion than **3B** at equilibrium. DFT now confirms that **2** and **4** are close in energy.

To further confirm this observation, an equimolar amount of pyridine and imidazole were simultaneously added to **2** to produce the corresponding equilibrium mixture of **2**, **3** and **4**. The resulting transmission constants agree with faster amine replacement in **2A** when compared to **2B** and faster rates of pyridine binding when compared to imidazole.

For dimethylsulfoxide, the corresponding complexes **5A** and **5B** result which now yield broad hydride resonances at *δ* –13.37 and *δ* –25.77 alongside sharp mutually coupled resonances at *δ* –21.14 and *δ* –25.89 (*J* = 9 Hz) respectively. The former hydride resonances of **5A** being broadened by rapid H_2_ loss. **5B** proved to exist in high proportion and fitted transmission rate constants confirm faster rates of amine replacement in **2A** (*k*_trans2A5A_ = 6 ± 1 × 10^–6^ s^–1^) when compared to **2B** and now a fast rate of isomerization of **5A** into **5B** (*k*_trans5A5B_ = 1 ± 0.1 × 10^–4^ s^–1^).

Upon removal of the H_2_ atmosphere and addition of ∼3 mL of degassed hexane to the equilibrium mixtures of **2** with **3**, **4**, or **5**, slow precipitation of a series of single crystals was observed. Subsequent X-ray diffraction studies revealed the common presence of known [Ir(amine)(η^2^-CO_3_)(IMes)(η^2^-imine)] as detailed in the ESI.† These are formed as the metal mediates the conversion of pyruvate into carbonate.^8^,^37^ It is the reversible binding of the amine, pyridine, imidazole, or DMSO that allows this conversion to occur.^37^

### Formation of novel iridium α-carboxyimine complexes **6–8** from **2** by variation of the coligand L

In contrast, while the addition of benzyl isocyanide does indeed result in the formation of **6A**, as reflected in the observation of hydride resonances at *δ* –8.49 and *δ* –24.69 (*J* = 5.5 Hz), this product is not stable over long time periods. The ratio of **2A** : **6A** changes from 1 : 2 to 1 : 6 after 5 min and 1 hour respectively. After this time products associated with the loss of imine are clearly detected. They yield two singlets in the hydride region at *δ* –10.22 and *δ* –12.34 in a 1.5 : 1 ratio which indicates the corresponding hydride ligands are *trans* to soft donors. According to accurate mass spectrometry analysis they reflect tris isocyanide and bis isocyanide-amine complexes. These products form in a 1 : 12 ratio when H_2_ is added to a solution of [IrCl(COD)(IMes)] with 5 equivalents PEA, and benzyl isocyanide. NMR characterization of this mixture confirms the singlet at *δ* –12.34 is due to tris isocyanide **6C** while the complex yielding the resonance at *δ* –10.22 could not be characterized due to its low concentration, but most likely corresponds to bis isocyanide-amine **6D**. As iridium(iii) isocyanide complexes have been previously reported the stability of these products is not surprising.^61^,^62^

The addition of a 4-fold excess of ethyl isothiocyanate to **2A** proceeds more rapidly to the related imine loss product [Ir(H)_2_(IMes)(phenethylamine)(SCNEt)_2_], **7C** than benzyl isocyanide yielding a single hydride signal at *δ* –16.05. At short reaction times resonances for **7A** at *δ* –17.87 and *δ* –28.27 (*J* = 9 Hz) are seen, although the ratio of **2A**, **2B**, **7A** and **7C** is 0 : 7.3 : 1 : 2.8 after just 30 min. There are also a wide range of reported stable isothiocyanate complexes.^63^,^64^ We note that **7C** is not formed when [IrCl(COD)(IMes)], PEA, and ethyl isothiocyanate react with H_2_. Full characterization data for **7C** is detailed in the ESI.† Hence the binding of ethyl isothiocyanate and benzyl isocyanide is sufficiently strong as to displace the chelated imine.

When the coligand is 4-chlorobenzenemethanethiol, the major product is **8C**. It yields a single hydride signal at *δ* –21.56 and reflects a species which is stable at 278 K for weeks. Structural characterization of this product by NMR spectroscopy confirmed it to be a monohydride with retained amine and carboxyimine ligands. It actually corresponds to the H_2_ replacement product that is formed by S–H bond oxidative addition. Such reactivity is well known for metal surfaces^65^ and bimetallic complexes.^66^,^67^ Similar iridium N-heterocyclic carbene complexes containing bound thiolates have been prepared from the displacement of Ir–Cl under basic conditions.^68^ Hence there is a clear rational for this behaviour and related products are expected to account for the catalyst deactivation seen in SABRE catalysis upon scavenging with a supported thiol.^55^,^69^

### Effect of coligand, L, on H_2_ exchange and hydride ligand NMR signal enhancements in **2–7**

**Fig. 3:**
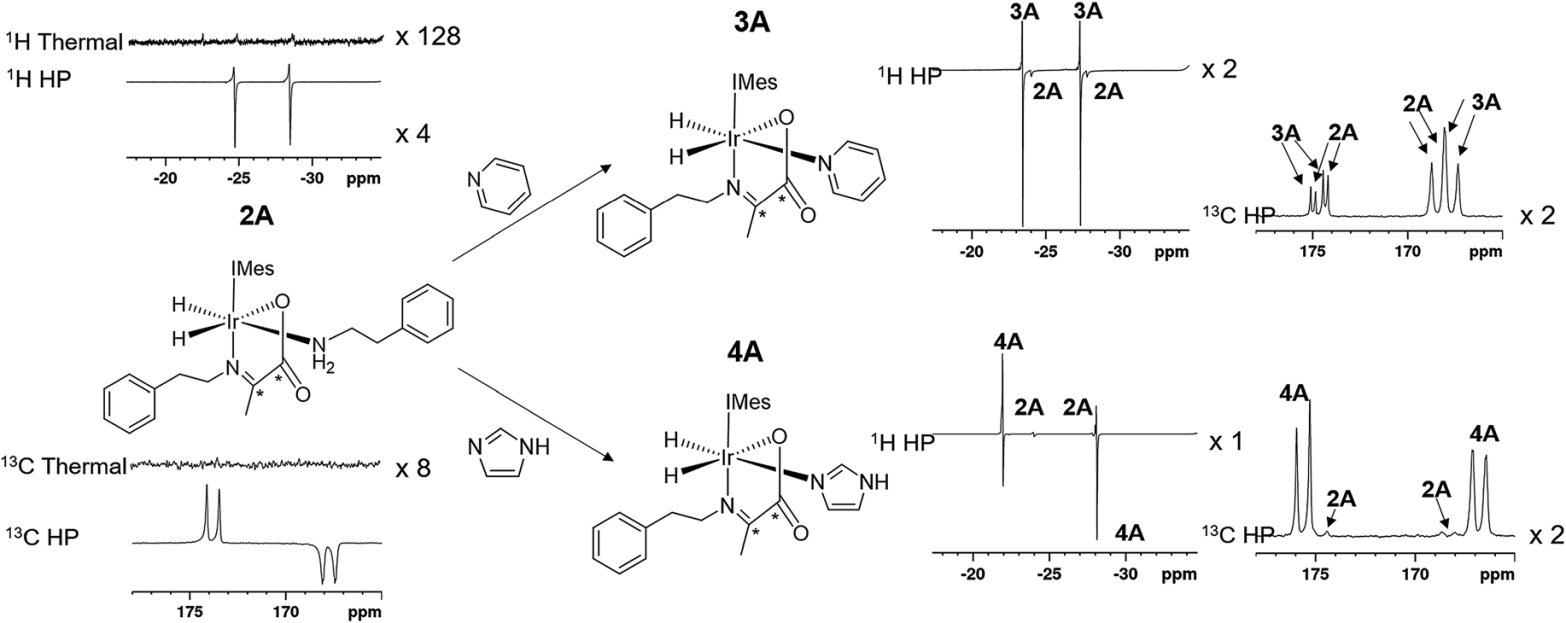
The structure and partial thermal and hyperpolarized ^1^H and ^13^C spectra for the amine complex **2A** are shown. Upon addition of the coligands pyridine or imidazole the complexes **3A** and **4A** form, the ^1^H and ^13^C resonances of which hyperpolarize in addition to those of the starting **2A** as shown. All ^1^H or ^13^C spectra are shown on the same vertical scale.

When an equilibrium mixture of **2** and **3** are shaken with *p*-H_2_ for 10 seconds at 65 G and then placed into the NMR spectrometer for detection with a 45° pulse, PHIP hyperpolarized hydride resonances are observed for both **2A** and **3A** as shown in Fig. 3. The hyperpolarized response of **3A** is ∼100 times more intense than that of **2A** which is now just 2% of the size that was observed before pyridine addition. These hydride signal enhancements are difficult to precisely quantify due to rapid dynamic exchange and peak overlap effects. Nonetheless, the selective radio frequency excitation of the hydride resonances of **3A** does reveal slow conversion to free H_2_ at a rate of 0.17 ± 0.01 s^–1^ at 273 K. This compares to the corresponding rate of H_2_ loss from **2A** of 1.14 ± 0.03 s^–1^ at 273 K and is consistent with the now proven higher thermodynamic stability of **3A**. The striking difference in hyperpolarization levels for **3A** might initially be thought to imply the opposite because they normally relate to the flux of *p*-H_2_ through a species. However, this behaviour can be rationalized by simply examining an equilibrium mixture of **2** and **4** with *p*-H_2_. As expected, hyperpolarized hydride resonances are observed for both **2A** and **4A**. The signal for **4A** is though just 16 times more intense than that of **2A** which has now dropped to 20% of its intensity prior to imidazole addition (Fig. 3). Significantly though, the corresponding EXSY measurements fail to detect any H_2_ loss for **4A** on this short relaxation controlled timescale. It is therefore clear that the high signal enhancements seen for **3A** and **4A** are not just due to H_2_ exchange within them without other ligand scrambling, termed direct-PHIP, but must also include a contribution from coligand addition to the common 5 coordinate reaction intermediate that results from amine loss from **2A**, a process that we now term indirect PHIP. The higher overall PHIP enhancements visible in equilibrium mixtures of **2** and **4** when compared to **2** and **3** are therefore due to proportionally more **2A** being present and hence greater turnover of the linked intermediate [Ir(H)_2_(IMes)(η^2^-α-carboxyimine)] which is trapped at steady state.

To further prove this hypothesis an equimolar addition of pyridine and imidazole was simultaneously made to a solution of **2**, forming an equilibrium mixture of **2**, **3** and **4** with amine, pyridine and imidazole coligands respectively. It might be expected from the faster rate of pyridine binding that the hyperpolarised response of **3A** would be greater than that of **4A**, but upon shaking with *p*-H_2_ the hydride resonances of these complexes hyperpolarise with a 1 : 2.8 : 4.8 intensity ratio of **2A**, **3A** and **4A** respectively. This discrepancy is simply due to the different extents to which polarization flows into the other NMR active sites in these metal complexes under SABRE. Polarization proves to be localised much more effectively on the hydride ligands of **4A** whereas in **3A** it readily spreads into the ^1^H sites of the pyridine coligand. This confirms that while these complexes form from the same common reaction intermediate, the relative rate constants for the formation of each cannot be measured directly from these intensity data as has been common for many other PHIP studies.

This deduction is confirmed by examining a solution of **2** containing equimolar amounts of pyridine-*d*_5_ and imidazole. The hyperpolarised hydride signals observed due to **2A**, **3A-*d*_5_** and **4A** now appear in a 1 : 6.4 : 4.6 ratio respectively. Hence **3A-*d*_5_** now exhibits a hydride signal of higher intensity than that of **4A** as the leakage of hyperpolarisation into the ^1^H resonances of the pyridine is quenched by deuteration. While these relative hyperpolarisation levels are now consistent with a faster rate of pyridine binding compared to imidazole, they still fail though to reflect the factor of 4 difference in observed transmission rates predicted earlier. One contribution to this difference will be the difference in average *T*_1_ values of the hydride ligands which are 3.5 s, 7.7 s, and 7.0 s for the hydride ligands of **2A**, **3A** and **4A** respectively at 9.4 T and will act to reduce the visible signal strengths seen for **2A** and **4A** relative to **3A**. However, the biggest challenge here arises from differing contributions from direct-PHIP and indirect-PHIP (coligand addition to the common 5 coordinate reaction intermediate) and what is known as internal resonance cancellation which reduces the measured intensity of antiphase peaks as a consequence of line-broadening effects. The width at half height for these three signals are 14, 11 and 10 Hz for **2A**, **3A-*d*_5_** and **4A** respectively which means this contribution will vary depending on the species.

When an equilibrium mixture of **2** and **5** is examined in a similar way, hyperpolarized responses are again seen for **2A** and **5A**, albeit this time the signal intensity of the **2A** response is half that which is seen prior to DMSO addition and the signals which corresponded to **5A** are actually incredibly weak. This is reflective of proportionally lower amounts of **2A** present at equilibrium, alongside a much greater proportion of PHIP inactive **5B**.

For similar reasons, equilibrium mixtures of **2** and **6** do not give any hyperpolarized ^1^H or ^13^C responses for any species. However, upon shaking mixtures of **2** and **7** with *p*-H_2_, weak hyperpolarized hydride resonances are seen for both **2A** and **7A**, although only at 9% and 3% of the initial signal intensity of **2A**. One further consequence of this dramatic reduction in *p*-H_2_ cycling is that no hyperpolarized ^13^C responses are observed.

### Isotopic labelling to improve ^13^C_2_ enhancements and singlet lifetimes of iridium α-carboxyimine complex **1**

**Chart 2:**
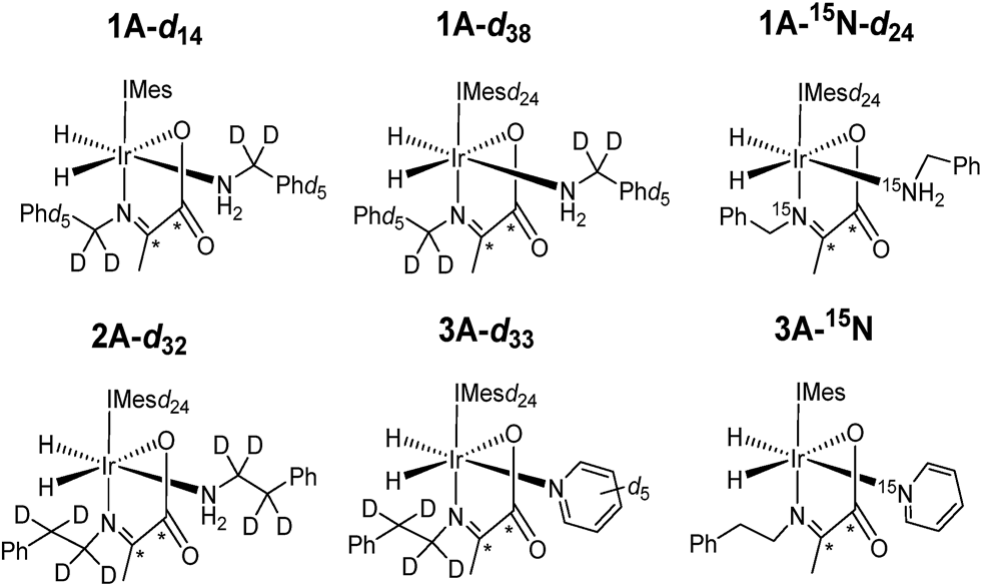
Structures of isotopologues used in this work.

Under SABRE the PHIP enhancement seen for the hydride ligands can transfer to coupled heteronuclear spins in a process that is magnetic field dependant.^26^ Furthermore, as ^13^C_2_ labelled pyruvate is used as a precursor, this transfer process could generate a singlet state in the product which is interesting because of its potentially long lifetime.^59^ Here we aim to probe these effects further and detail how coupled spins in L may influence this outcome. In fact, it has already been reported that the *p*-H_2_ derived singlet order in **1A** and **2A** can transfer into the ^13^C_2_ spins of the carboxyimine core.^37^ For SABRE, relaxation of a substrate when it is bound to the catalyst limits the degree of hyperpolarisation that can be created in the free ligand. This effect can be reduced by the inclusion of deuterium labels with the NHC ligand as this acts to increase bound substrate relaxation times and the visible lifetime of hyperpolarized signals.^26^,^40^,^56^,^57^ Hence we link these approaches here to create the ^2^H labelled analogues **1A-*d*_14_** and **1A-*d*_38_** of Chart 2 and test their hyperpolarisation properties. As expected, these complexes react with *p*-H_2_ to yield good ^13^C_2_ signal enhancements in the corresponding ^13^C NMR experiments (Table 3). Furthermore, a singlet state lifetime of 19.9 ± 1.0 s (compared to 10.9 ± 1.1 s in the ^1^H form) for **1A-*d*_38_** was determined. In contrast **1A-*d*_14_**, where the starting amine is deuterated, exhibits a similar lifetime to that of **1A** (7.9 ± 0.9 s). Hence we can conclude interactions with the carbene ligand are critical to the resulting spin state lifetime.

**Table 3:** ^1^H and ^13^C signal enhancements (*ε*) and ^13^C_2_ singlet lifetimes of deuterated analogues

Complex	*ε* ^1^H hydride/fold	*ε* ^13^C_2_ imine/fold	^13^C_2_ singlet lifetime/s
**1A-*d*_14_**	110	510	7.9 ± 0.9
**1A-*d*_38_**	300	560	19.9 ± 1.0
**1A-^15^N-*d*_24_**	480	750	17.5 ± 3.9
**2A-*d*_32_**	740	340	N/A
**2A**/**3A**	N/A	**2A**, 220	N/A
		**3A**, 190	
**2A-*d*_32_**/**3A-*d*_33_**	**2A-*d*_32_**, 720	**2A-*d*_32_**, 330	N/A
	**3A-*d*_33_**, 380	**3A-*d*_33_**, 260	
		Pyridine, 0	
**2A/3A-^15^N**	**2A**, 230	**2A**, 230	N/A
	**3A-^15^N**, 390	**3A-^15^N**, 260	
		Pyridine, 190	
**1A-^15^N-*d*_24_**/**3A-^15^N_2_-*d*_24_**	**1A-^15^N-*d*_24_**, 1620	**2A** N/A^*a*^	N/A
	**3A-^15^N_2_-*d*_24_**, 590	**3A-^15^N**, N/A^*a*^	
		Pyridine, 870	

^*a*^Spectral overlap prevents signal enhancements being calculated for each complex. Note pyridine enhancements include bound and free signals.

It has previously been suggested that ^13^C hyperpolarization levels can be increased by removing the effect of quadrupolar ^14^N nuclei in related systems.^46^ Therefore, a **1A-^15^N-*d*_24_** isotopologue in which ^15^N-labeled benzylamine and IMes-*d*_24_ were used as precursors was also prepared. The resulting ^13^C_2_ NMR signal enhancements and magnetic state lifetime for this complex are now 750 fold and 17.5 ± 3.9 s respectively. This suggests that while the presence of ^15^N does indeed enhance the level of ^13^C polarization, it does not have a large effect on singlet state lifetime here. For this complex, a SABRE-SHEATH measurement was also undertaken to hyperpolarize the ^15^N responses, this revealed strong signals for the bound imine and free amine, as detailed in the ESI.†

### Effect of coligand, L, on ^13^C_2_ enhancement levels and singlet spin order lifetime

**Fig. 4:**
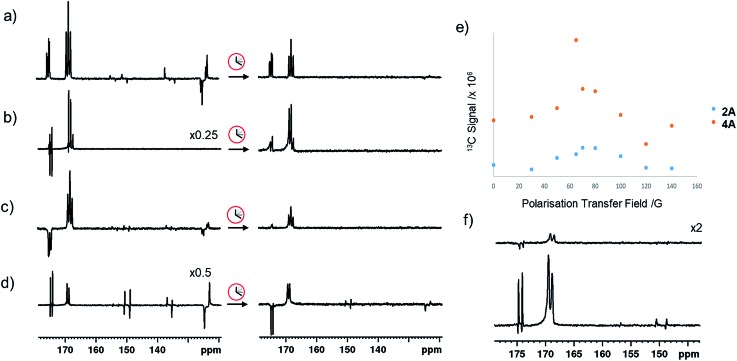
Partial hyperpolarized ^13^C spectra for equilibrium mixture of (a) **2A** and **3A**, (b) **2A-*d*_32_** and **3A-*d*_33_**, (c) **3A** and **3A-^15^N** and (d) **1A-^15^N-*d*_24_** and **3A-^15^N_2_-*d*_24_** shaken for 10 seconds at 65 G (left) and after storing in a mu metal shield for 5 seconds after 65 G shaking (right) (e) integrated hyperpolarized resonances for **4A** and **2A** as a function of polarization transfer field (f) hyperpolarized ^13^C responses for an equilibrium mixture of **1A-^15^N-*d*_24_** and **3A-^15^N_2_-*d*_24_** shaken for 10 seconds at 65 G and then stored in a mu metal shield for 30 seconds (top) and shaking in the shield for 5 seconds after 30 seconds storage time.

In the corresponding ^13^C experiments with added coligand, hyperpolarized responses for the ^13^C_2_ labelled imine cores of **3A** and **4A** are readily visible at *δ* 174.79 and *δ* 167.71 (*J* = 66.5 Hz) and *δ* 175.63 and *δ* 166.76 (*J* = 66.5 Hz) respectively. These resonances partially overlap with those of **2A** and appear with similar intensity in the case of **3A** but are 17 times stronger in the case of **4A** as shown in Fig. 4. The hyperpolarized ^13^C_2_ resonances of **3A** and **4A** though no longer exhibit the original ‘up–up–down–down’ pattern typical of the singlet state.^37^ This suggests that Zeeman magnetization is now dominant and that rapid singlet state decoherence occurs. In fact, shaking samples of **4A** with *p*-H_2_ at different polarization transfer fields yield a hyperpolarized ^13^C_2_ response between 1 mG and 100 G, although the maximum signal intensity is seen at 65 G as shown in Fig. 4e. The spin states detected in these complexes can be the result of *p*-H_2_ derived transfer within **3A** or **4A**, or from *p*-H_2_ transfer within **2A** and subsequent ligand loss and binding of the co-ligand.

There are three hyperpolarization *p*-H_2_ derived proton transfer mechanisms that might operate in these complexes to enhance the signals of these ^13^C nuclei and their efficiencies must be linked to the ligand exchange dynamics. The first of these transfer processes, *R*_1_, is singlet magnetization transfer from *p*-H_2_, and its efficiency should be independent of magnetic field.^59^ The second, *R*_2_ is direct polarization transfer to create ^13^C Zeeman magnetization in a process known as SABRE-SHEATH that occurs at a mG field. Finally, Zeeman magnetization can also be relayed indirectly from the hydride ligands to ^13^C *via* hyperpolarized ^1^H sites in process, *R*_3_, whose first step will be optimal at around 65 G.^26^,^39^,^56^

Previous work has shown that when mixtures containing solely **1** or **2** are shaken at 65 G, the resonance condition for *R*_1_ is met alongside *R*_3_ and long lived singlet state profiles dominate as previously reported.^37^ The maintenance of this singlet in **1A** and **2A** is augmented during this process by on-going *p*-H_2_ exchange, this effect is expected to be minimal in **3A** and **4A** due to their much slower H_2_ loss rates.

When mixtures of **2** and **3** or **2** and **4** are shaken with *p*-H_2_ at 65 G the ^13^C response of **2A** is dramatically reduced in the same way as their ^1^H hydride signals. The corresponding ^13^C signals for **3A** and **4A** appear as a mixture of both singlet and Zeeman magnetization as shown in Fig. 4a. The role played by **2A** in the formation of **3A** and **4A** therefore results in singlet decoherence.

As deuterium labelling can enhance singlet state lifetimes and perhaps suppress singlet decoherence, we tested the effect of its incorporation on the spin-state lifetime of **3A** by using phenethylamine-*d*_4_, IMes-*d*_24_, and pyridine-*d*_5_ to create an equilibrium mixture of **2A-*d*_32_** and **3A-*d*_33_**. The resulting ^13^C_2_ signals of both **2A-*d*_32_** and **3A-*d*_33_** after transfer at 65 G proved to be much stronger than their *protio*-analogues, as shown in Fig. 4b. Now, the balance in Zeeman and singlet magnetization (*R*_3_ and *R*_1_ processes) does indeed favour the latter and unusual ^13^C signal behaviour is discerned. This is in part a consequence of the fact polarization no longer spreads into the coligand/catalyst. However, ^2^H labelling suppresses the indirect polarization transfer route *R*_3_ which is *via*^1^H and must occur though the CH_3_ group of the imine or the NH_2_ group of the amine. Consequently, the singlet spin order of **3A-*d*_33_** does not decohere rapidly. We repeated this process at 65 G before introducing a 5 second storage period in a mu metal shield before observation. The resulting signals for **3A-*d*_33_** then lose much of their singlet character due to decoherence effects.

Synthesizing **3A** with ^15^N-labeled pyridine to create **3A-^15^N** results in a ^13^C_2_ profile that is more typical of a singlet state, as shown in Fig. 4c. We demonstrated previously that while the introduction of this ^15^N label in **1A-^15^N-*d*_24_** did increase signal strength, it did not significantly enhance the singlet state lifetime of **1A**. Now though, it significantly increases the singlet state retention in **3A-^15^N** and confirms quadrupolar ^14^N plays a major role in its relaxation.

When **3A-^15^N_2_-*d*_24_** was studied, in which both pyridine and benzylamine precursors were ^15^N labelled and the IMes catalyst was deuterated, the hyperpolarized profile after 10 second shaking at 65 G shown in Fig. 4d was obtained. However, storage at this point in a mu metal shield for 10 seconds instead of proceeding directly to data collection now allows the singlet state product profile to be readily distinguished. Consequently, full ^15^N labelling clearly extends the lifetime over which this product remains visible. However, when **3A-^15^N_2_-*d*_24_** is first polarised for 10 seconds at 65 G before being left for 30 seconds in a mu metal shield before again shaking it for 5 seconds now in the shield (Fig. 4f) the measured signals are due predominantly to low field direct SABRE-SHEATH transfer (*R*_2_) and outweigh any singlet state that remains.

### Heteronuclear coligand enhancements of iridium α-carboxyimine complexes

The hyperpolarized ^13^C resonances of bound and free pyridine that are also observed in **3A**, as shown in Fig. 4, are also worthy of comment. The significant 866-fold total ^13^C enhancement of pyridine suggests that the incorporation of a chelating carboxyimine may reflect a positive route to increasing substrate polarization. In contrast no ^13^C signals of the amine of **2A** or the imidazole of **4A** are visible. This suggests that the lifetime of pyridine in **3A** is suitable for it to receive polarization from its hydride ligands whereas that of the amine is too short. For ^15^N pyridine, the corresponding ^1^H NMR total signal gains seen under these conditions are 1600-fold while for imidazole they are just 31-fold.

Interestingly, the hyperpolarized ^13^C response for pyridine vanishes for **3A-*d*_33_**, as shown in Fig. 4c. This confirms that under 65 G transfer the main route to ^13^C signal gain is *via* the pyridine proton sites and any direct transfer, or indeed transfer *via* nitrogen is less important. **3A-^15^N** does yield hyperpolarized ^13^C pyridine responses but they are of lower intensity than those in **3A**, as shown in Fig. 4d. Hence, at this polarization transfer field there is no significant polarization relay *via*^15^N.^70^

## Experimental

All NMR measurements were carried out on a 400 MHz Bruker Avance III spectrometer at 298 K unless otherwise stated. *para*-Hydrogen (*p*-H_2_) was produced by passing hydrogen gas over a spin-exchange catalyst (Fe_2_O_3_) at 28 K and used for all hyperpolarization experiments. This method produces constant *p*-H_2_ with *ca.* 93% purity. ^1^H (400 MHz) and ^13^C (100.6 MHz) NMR spectra were recorded with an internal deuterium lock. Chemical shifts are quoted as parts per million and referenced to CD_2_Cl_2_. ^13^C NMR spectra were recorded with broadband proton decoupling. Coupling constants (*J*) are quoted in hertz. Electrospray high and low resolution mass spectra were recorded on a Bruker Daltronics microOTOF spectrometer. The coligands pyridine, imidazole, thiophene, acetonitrile, DMSO, benzyl isocyanide, ethylisothiocyanate and 4-chlorobenzenemethanethiol were all purchased from Sigma Aldrich, Fluorochem or Alfa-Aesar and used as supplied without further purification.

The shake & drop method was employed for recording hyperpolarized SABRE NMR spectra.^26^ Samples were prepared in a 5 mm NMR tube that was fitted with a J. Young's tap. The iridium precatalyst used was [IrCl(COD)(IMes)] (where IMes = 1,3-bis(2,4,6-trimethyl-phenyl)imidazole-2-ylidene and COD = *cis*,*cis*-1,5-cyclooctadiene) and was synthesized in our laboratory according to a literature procedure.^71^ The NMR samples were subsequently degassed by two freeze–pump–thaw cycles before filling the tube with *p*-H_2_ at 3 bar pressure. Once filled with *p*-H_2_, the tubes were shaken vigorously for 10 seconds in the 65 Gauss fringe field of a 9.4 T Bruker spectrometer. Immediately after that, the NMR tubes were put inside the spectrometer for NMR detection. ^1^H shake and drop measurements were recorded with a 45° pulse unless otherwise stated.

Hydride signal enhancements were calculated by dividing the hyperpolarized integral intensity by the corresponding intensity from a 1 scan thermal recorded and processed under the same conditions. Thermal 1,2–^13^C_2_ coordinated imine resonances were not visible in 1 thermal scan, so ^13^C enhancements were calculated as shown in the ESI.† Hydrogen exchange rates and singlet state lifetimes were recorded as previously reported.^37^ All characterisation data, kinetic modelling and DFT calculations are shown in the ESI.†

### Formation of **1** and **2**

3 bar hydrogen gas was added to a degassed solution of [IrCl(COD)(IMes)] (2 mg, 0.003 mmol; 1 equivalent) and BnNH_2_ or phenethylamine (PEA) (1.8 μL or 2.0 μL, 0.015 mmol, 5 equivalents) (for **1** and **2** respectively) dissolved in 0.6 mL DCM-*d*_2_. Upon the formation of [Ir(H)_2_(IMes)(NH_2_Bn)_3_] the solution goes from yellow to colourless.^39^ At this point sodium pyruvate-1,2-[^13^C_2_] (1.8 mg, 0.015 mmol, 5 equivalents) was dissolved in 40 μL H_2_O and added to the NMR tube under a flow of N_2_. The tube was repressurized with 3 bar *p*-H_2_ and left overnight to allow the formation of an equilibrium mixture of **1** or **2** as previously reported.^37^

### Formation of **3–8**

1 μL (∼5 equivalents relative to precatalyst) of the corresponding coligand (pyridine for **3**, DMSO for **5**, benzylisocyanide for **6**, ethylisothiocyanate for **7** and 4-chlorobenzenemethanethiol for **8**) was added to **2** under a flow of N_2_ gas before the NMR tube was repressurised with 3 bar hydrogen gas. **4** was formed from the addition of 2 mg imidazole in 40 μL DCM-*d*_2_ to **2** in the same manner.

## Conclusions

In conclusion, we have synthesised a range of novel [Ir(H)_2_(IMes)(α-^13^C_2_-carboxyimine)L] complexes in which the identity of the coligand L can be amine, pyridine, DMSO, benzyl isothiocyanide or ethyl isothiocyanate. In the latter two cases further reaction and sample degradation occurs to yield new products that include [Ir(H)_2_(IMes)(EtSCN)_2_(amine)]. Upon the addition of a thiol we observe and characterise the novel SH bond activated [Ir(H)(IMes)(α-^13^C_2_-carboxyimine)(S-thiolate)] product. When examined with *para*-hydrogen, complexes in which L is amine, pyridine or imidazole show significant ^1^H hydride and ^13^C_2_ imine signal enhancements. We have shown that dissociative amine loss is a key step in the *p*-H_2_ exchange process that leads to these signal enhancements. The coligands effectively trap the associated hyperpolarised intermediate [Ir(H)_2_(IMes)(α-^13^C_2_-carboxyimine)] to achieve this result. Despite this mechanism, the hyperpolarised hydride signal intensities are not always reflective of the rates of coligand binding to this intermediate. This is because hydride based hyperpolarisation flows into the ligands attached to the complex and therefore great care must be taken when attempting to interpret such signal intensity data in a quantitative fashion.

This study has also demonstrated how isotopic labelling techniques can be used to achieve ^13^C_2_ signal enhancement levels of 750-fold whilst accessing singlet state lifetimes of up to 20 seconds. Coligand addition can though cause rapid decoherence of any resulting ^13^C_2_ singlet order in these products, but it can be preserved to some extent by prudent isotopic labelling. Furthermore, the α-carboxyimine ligand acts to block exchangeable coordination sites with the result that significant ^13^C enhancement can be seen in pyridine as *p*-H_2_ hyperpolarisation is now directed efficiently into the co-ligand.

The strongly enhanced hydride resonances of the array of [Ir(H)_2_(IMes)(α-^13^C_2_-carboxyimine)L] complexes provide a unique response which means they can act as efficient sensors of the identity of L. Given this spectral region is normally transparent to ^1^H NMR background signals the detection of trace compounds through these responses might subsequently reflect a key application of this work.

## Conflicts of interest

There are no conflicts to declare.

## Supplementary Material

Supplementary information

Crystal structure data
