# Supplementary material for: Using coligands to gain mechanistic insight into iridium complexes hyperpolarized with para-hydrogen
Source: Chem Sci. 2019 Mar 19;10(20):5235–45. doi: 10.1039/c9sc00444k (PMC6540910; doi:10.1039/c9sc00444k)
Supplement: Supplementary file 1 [file SC-010-C9SC00444K-s001.pdf]

Electronic Supplementary Material (ESI) for Chemical Science.
This journal is © The Royal Society of Chemistry 2019

Supporting Information

Using coligands to gain mechanistic insight into iridium complexes hyperpolarized with *para*-hydrogen

Ben. J. Tickner, Richard O. John, Soumya S. Roy, Sam J. Hart, Adrian C. Whitwood and Simon B. Duckett*

Table of Contents

S1: General remarks
S2: Experimental procedures
S3: Characterization of products
- S3.1: Characterization of **4A**
- S3.2: Characterization of **5**
- S3.3: Characterization of **6**
- S3.4: Characterization of **7C**
- S3.5: Characterization of **8C**
- S3.6: X-ray crystallography of **3**, **4** and **5**

S4: Density Functional Theory (DFT) calculations
- S4.1 General remarks
- S4.2 Optimised geometry of the 5 coordinate intermediate
- S4.3 DFT results

S5: Kinetic modelling and rate constants
- S5.1 Kinetic modelling and rate constants for formation of **3**-**5** from **2**
- S5.2 Kinetic modelling and rate constants for formation of **3** and **4** from **2**

S6: Hydrogen exchange rates
S7: ^{15}N Hyperpolarisation studies
S8: Hyperpolarized singlet decay measurements

S1: General remarks

All NMR measurements were carried out on a 400 MHz Bruker Avance III spectrometer at 298 K unless otherwise stated. *Para*-hydrogen (p-H_2) was produced by passing hydrogen gas over a spin-exchange catalyst (Fe_2O_3) at 28 K and used for all hyperpolarization experiments. This method produces constant p-H_2 with ca. 93% purity. ^1H (400 MHz) and ^{13}C (100.6 MHz) NMR spectra were recorded with an internal deuterium lock. Chemical shifts are quoted as parts per million and referenced to CD_2Cl_2. ^{13}C NMR spectra were recorded with broadband proton decoupling. Coupling constants (J) are quoted in Hertz. Electrospray high and low resolution mass spectra were recorded on a Bruker Daltronics microOTOF spectrometer. The coligands pyridine, imidazole, thiophene, acetonitrile, DMSO, benzyl isocyanide, ethylisothiocyanate and 4-chlorobenzenemethanethiol were all purchased from Sigma Aldrich, Fluorochem or Alfa-Aesar and used as supplied without further purification.

<u>Shake and drop method</u>

The shake & drop method was employed for recording hyperpolarized SABRE NMR spectra.[1] Samples were prepared in a 5 mm NMR tube that was fitted with a J. Young's tap. The iridium precatalyst used was [IrCl(COD)(IMes)] (where IMes = 1,3-bis(2,4,6-trimethyl-phenyl)imidazole-2-ylidene and COD = cis,cis-1,5-cyclooctadiene) and was synthesized in our laboratory according to a literature procedure.[2] The NMR samples were subsequently degassed by two freeze-pump-thaw cycles before filling the tube with p-H_2 at 3 bar pressure. Once filled with p-H_2, the tubes were shaken vigorously for 10 seconds in the 65 Gauss fringe field of a 9.4 T Bruker spectrometer. Immediately after that, the NMR tubes were put inside the spectrometer for NMR detection. ^1H shake and drop measurements were recorded with a 45° pulse unless otherwise stated.

<u>Signal enhancements</u>

Signal enhancements were calculated by comparing hyperpolarized spectra its thermal counterpart as described below. Both hyperpolarized and thermally polarized spectra were recorded on the same sample using the same spectrometer settings.

Hydride signal enhancements were calculated by dividing the hyperpolarized integral intensity by the corresponding intensity from a 1 scan thermal recorded and processed under the same conditions. Thermal 1,2-$^{13}C_2$ coordinated imine resonances were not visible in 1 thermal scan, so ^{13}C enhancements, ε_{13C2}, were estimated using equation 1 where S_{Hy} is the hyperpolarized ^{13}C signal intensity of both $^{13}C_2$ resonances of the complex, Mr_{cat} and M_{cat} are the molecular mass and mass of iridium precatalyst used, S_{ref}, M_{ref} and Mr_{ref} are the thermal ^{13}C signal intensity, mass and molecular mass of a $^{13}C_2$ containing reference sample, which here was sodium-1,2-pyruvate-[$^{13}C_2$].

$$\varepsilon_{13C2} = \frac{S_{Hy} Mr_{cat} M_{ref}}{M_{cat} S_{Ref} R_{Min} Mr_{ref}} \qquad (1)$$

Pyridine ^{13}C Enhancements $\varepsilon_{13C(Py)}$ were calculated using equation 2 where S_{Hy} is the total hyperpolarized pyridine (free and bound) signals, Mr_{Py}, D_{py} and V_{Py} are the molecular weight, density and volume of pyridine respectively, V_{sol}, D_{sol} and Mr_{sol} is the volume, density and molecular weight of the DCM solvent used and S_{sol} is the DCM ^{13}C signal from a 1 scan thermal.

$$\varepsilon_{13C(Py)} = \frac{S_{Hy} Mr_{Py} V_{sol} D_{sol}}{5 D_{py} V_{Py} S_{sol} Mr_{sol}} \qquad (2)$$

S2: Experimental procedures

Formation of 1 and 2: 3-bar hydrogen gas was added to a degassed solution of [IrCl(COD)(IMes)] (2 mg, 0.003 mmol; 1 equivalent) and $BnNH_2$ or Phenethylamine (PEA) (1.8 µL or 2.0 µL , 0.015 mmol, 5 equivalents) (for **1** and **2** respectively) dissolved in 0.6 mL DCM-d_2. Upon the formation of [Ir(H)2(IMes)(NH_2Bn_3)] the solution goes from yellow to colourless.[3] At this point sodium pyruvate-1,2-[$^{13}C_2$] (1.8 mg, 0.015 mmol, 5 equivalents) was dissolved in 40 µL H_2O and added to the NMR tube under a flow of N_2. The tube was repressurized with 3 bar p-H_2 and left overnight to allow the formation of an equilibrium mixture of **1** or **2** as previously reported.[4]

Formation of 3-8: 1 µL (~5 equivalents relative to precatalyst) of the corresponding coligand (pyridine for **3**, DMSO for **5**, benzylisocyanide for **6**, ethylisothiocyanate for **7** and 4-chlorobenzenemethanethiol for **8**) was added to **2** under a flow of N_2 gas before the NMR tube was repressurized with 3-bar hydrogen gas. 4 was formed from the addition of 2 mg imidazole in 40 µL DCM-d_2 to **2** in the same manner.

S3 Characterization of products

S3.1: Characterization of 4A

An equilibrium mixture of **2** and **4** was formed according to S2. ~3 mL degassed hexane was added to cause the precipitation of **4A** as a bright orange solid which was isolated by filtration and drying under vacuum. Samples were taken for MS analysis and the remainder was redissolved for NMR characterization at 245 K.

HR-ESI$^+$/MS (m/z): for $[C_{38}{}^{13}C_2H_{15}D_{24}IrN_5O_2 - H_3]^+$, calcd 780.4301, found 780.4338; for $[C_{38}{}^{13}C_2H_{25}D_{24}IrN_4O_2]^+$, calcd 836.5058, found 836.4362; for $[C_{30}{}^{13}C_2H_{12}D_{24}IrN_3O_2]^+$, calcd 713.4010, found 713.4030.

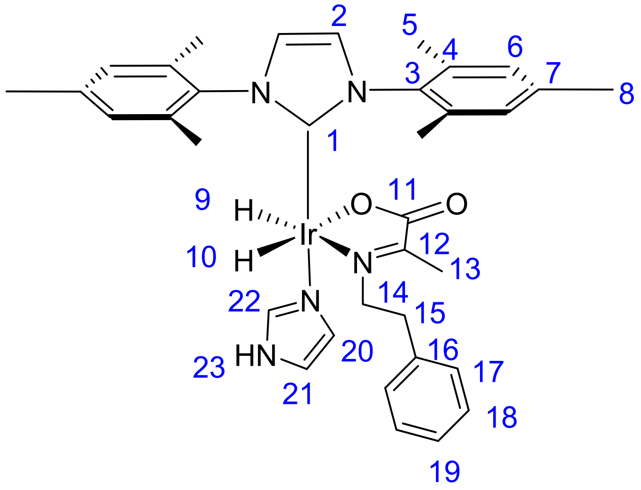

Figure S1) Structure of **4A** determined from the NMR data given in Table S1.

Table S1) NMR data collected at 245 K used to determine the structure of **4A** shown in Figure S1.

Resonance	1H	13C
1	-	N/A (~ 141)
2	6.84, 6.86	128.06, 128.17
3	-	138.10
4	-	N/A (~ 137)
5	2.19	~ 18.6 (overlap)
6	7.02	128.87
7	-	N/A (~ 137)
8	~ 2.2 (overlap)	~ 22.5 (overlap)
9	− 21.63 (d, 2JHH = 8 Hz)	-
10	− 28.25 (dd, 2JHH = 8 Hz, J = 5 Hz)	-
11	-	175.86 (d, 1JCH = 67 Hz)
12	-	166.28 (d, 1JCH = 67 Hz)
13	1.62	16.32
14	3.75, 3.90	43.72
15	2.78, 2.92	40.13
16	-	139.50
17	7.25	128.32
18	7.23	128.95
19	7.18	126.15
20/21	6.49, 6.69	132.68, 135.84
22	6.78	115.99
23	8.33	-

S3.2: Characterization of 5

An equilibrium mixture of **2** and **5** was formed according to S2. NMR characterization was not performed due to the presence of multiple species in solution causing spectral crowding and peak overlap. HR-ESI$^+$/MS (m/z): for **5** [$C_{34}H_{45}IrN_3O_3S$ + H]$^+$, calcd 768.2811, found 768.2815; for [$C_{34}H_{45}IrN_3O_3S$ + Na]$^+$, calcd 790.2630, found 790.2635; for [$C_{34}H_{45}IrN_3O_3S$ – C_2H_7OS]$^+$, calcd 688.2515, found 688.2528; [$C_{34}H_{45}IrN_3O_3S$ – C_2H_6OS + Na]$^+$, calcd 712.2491, found 712.2483.

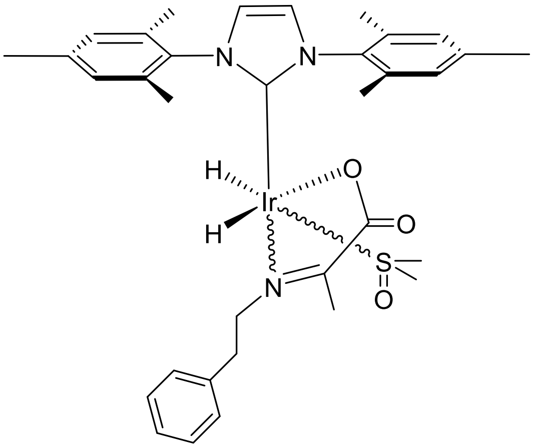

Figure S2) Structure of **5** determined from the MS data.

S3.3: Characterization of 6

6A was formed according to S2 and characterized using NMR spectroscopy at 245 K.

HR-ESI+/MS (m/z): for **6A** [$C_{40}H_{45}IrN_4O_2$ + H]+, calcd 807.3250, found 807.3240; for [$C_{40}H_{45}IrN_4O_2$ + Na]+, calcd 829.3069, found 829.3057; for **6C** [$C_{45}H_{46}IrN_5$]+, calcd 850.3461, found 850.3452; for **6D** [$C_{45}H_{51}IrN_5$]+, calcd 854.3714, found 854.3765;

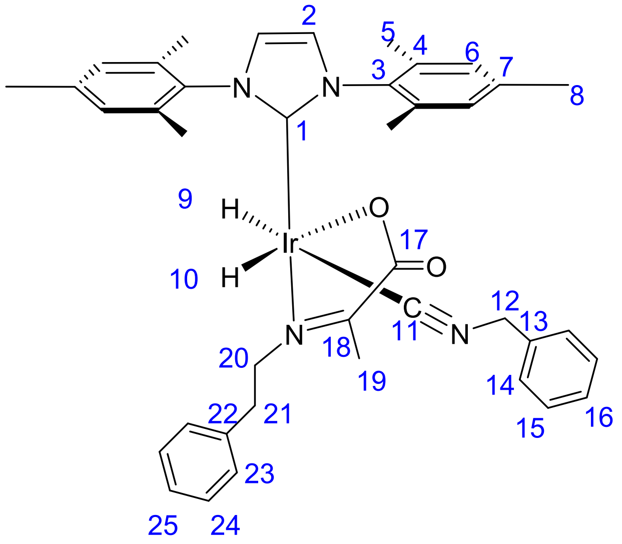

Figure S3) Structure of **6A** determined from the NMR data given in Table S2.

Table S2) NMR data collected at 245 K used to determine the structure of **6A** shown in Figure S3.

Resonance	^{1}H	^{13}C
1	-	140.27
2	7.33	128.39
3	-	138.62
4	-	136.43/136.46
5	2.10/2.17	18.13/18.18
6	6.94/7.01	128.44/128.50
7	-	138.09
8	2.29	21.09
9	− 8.57	-
10	− 24.63	-
11	-	153.6
12	2.13	121.4
13	-	N/A (~130 overlap)
14	6.96	128.93
15	7.18	128.29
16	N/A (~7.2 overlap)	N/A (~128 overlap)
17	173.7	-
18	169.22	-
19	1.67	16.56
20	3.72, 3.87	63.69
21	2.52	35.39
22	-	N/A (~130 overlap)
23	6.98	126.41
24	N/A (~7.2 overlap)	N/A (~128 overlap)
25	N/A (~7.2 overlap)	N/A (~128 overlap)

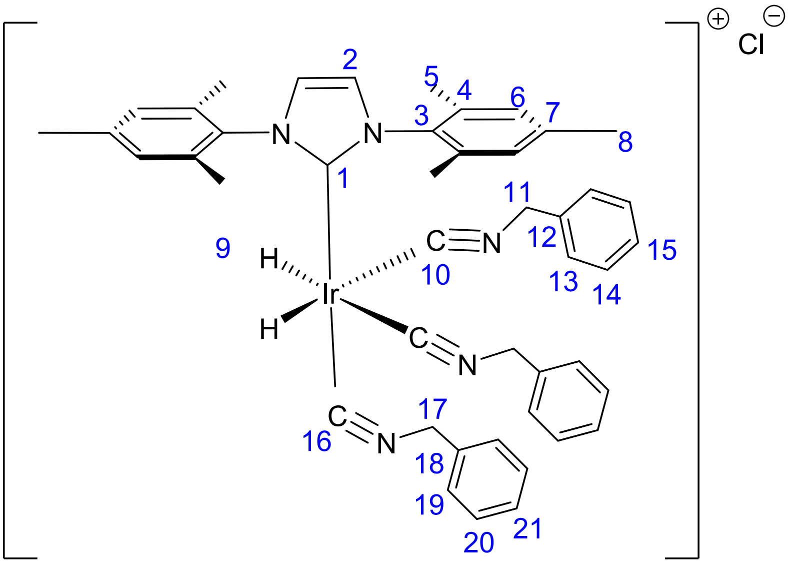

Figure S4) Structure of **6C** determined from the NMR data given in Table S3.

Table S3) NMR data collected at 245 K used to determine the structure of **6C** shown in Figure S4.

Resonance	^{1}H	^{13}C
1	-	N/A ~140
2	7.08	123.02
3	-	135.57
4	-	137.24
5	2.00	18.40
6	6.98	129.06
7	-	139.42
8	2.28	20.9
9	− 12.34	-
10	-	156.90
11	4.73	117.93
12	-	N/A (~130 overlap)
13	7.17	N/A (~128 overlap)
14	N/A (~7.2 overlap)	N/A (~128 overlap)
15	N/A (~7.2 overlap)	N/A (~128 overlap)
16	-	150.78
17	4.82	113.95
18	-	N/A (~130 overlap)
19	7.12	N/A (~128 overlap)
20	7.23	N/A (~128 overlap)
21	N/A (~7.2 overlap)	N/A (~128 overlap)

S3.4: Characterization of 7C

7C was formed according to S2 and characterized using NMR spectroscopy at 245 K.

HR-ESI⁺/MS (m/z): for $[C_{36}H_{51}IrN_5S_2 – C_3H_8NS + Na]^+$, calcd 743.2735, found 743.2923.

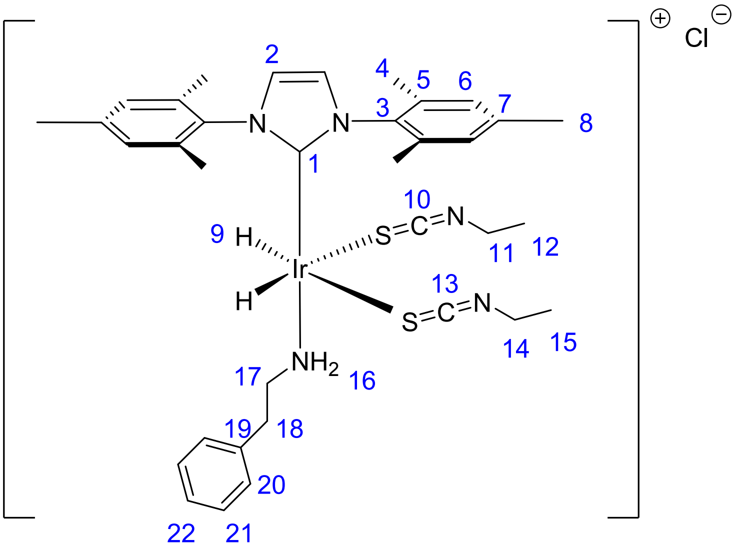

Figure S5) Structure of **7C** determined from the NMR data given in Table S4.

Table S4) NMR data collected at 245 K used to determine the structure of **7C** shown in Figure S5

Resonance	^1H	^{13}C
1	-	140.38
2	~ 7.2 (overlap)	N/A (~126)
3	-	138.23
4	2.13/2.16	17.64/17.67
5	-	135.53
6	7.12/7.19	128.58/129.61
7	-	137.77
8	2.40	21.21
9	– 16.05	-
10/13	-	119.99/120.28
11/14	3.57/3.59	39.94/40.27
12/15	1.20/1.35	15.25/15.46
16	1.80	-
17	3.85	35.05
18	2.04, 2.31	28.98
19	-	~130 (overlap)
20	6.96	128.72
21	7.23	~128-130 (overlap)
22	~7.2 (overlap)	~128-130 (overlap)

S3.5: Characterization of 8C

8C was formed according to S2 and characterized using NMR spectroscopy at 245 K.

HR-ESI+/MS (m/z): for [$C_{45}{}^{13}C_2H_{54}ClIrN_4O_2S$ + H]+, calcd 969.3431, found 969.3385; for [$C_{45}{}^{13}C_2H_{54}ClIrN_4O_2S$ −H_6 + Na]+, calcd 985.2781, found 985.3394; for [$C_{45}{}^{13}C_2H_{54}ClIrN_4O_2S$ −$C_7H_{11}ClS$]+, calcd 806.3082, found 806.2784.

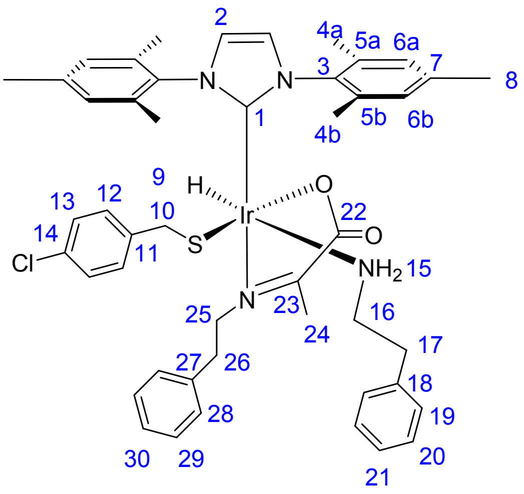

Figure S6) Structure of **8C** determined from the NMR data given in Table S5.

Table S5) NMR data collected at 245 K used to determine the structure of **8C** shown in Figure S6.

Resonance	^{1}H	^{13}C
1	-	140.48
2	6.76	121.67
3	-	~133 (overlap)
4a/4b	2.18/2.13	18.42/17.65
5a/5b	-	128.08/128.63
6a/6b	7.12/7.04	133.72/133.61
7	-	136.88
8	2.37	21.18
9	− 21.58	-
10	3.18	47.88
11	-	130.63
12	7.17	128.02
13	~7.2 (overlap)	~128 (overlap)
14	-	146.86
15	1.88, 2.08	-
16	2.22, 2.25	30.58
17	3.13, 3.16	35.23
18	-	138.02
19	6.86	130.52
20	7.22	128.83
21	7.20	126.52
22	-	175.51
23	-	167.86
24	1.56	16.20
25	3.49, 3.53	25.54
26	2.91, 2.96	17.83
27	-	135.50
28	7.21	127.73
29	~7.2 (overlap)	~128 (overlap)
30	~7.2 (overlap)	~128 (overlap)

S3.6: X-ray crystallography of 3 and 6

Crystals were prepared by removing the H_2 atmosphere, concentrating a sample to ~0.2 mL in a stream of N_2 gas, then layering ~3 mL degassed hexane slowly on top of the remaining solution in the NMR tube and leaving it under N_2 for period of several weeks. A suitable crystal was selected and mounted on an Oxford Diffraction SuperNova- X-ray diffractometer. The crystal was kept at 110 K during data collection. Diffractometer control, data collection, initial unit cell determination, frame integration and unit-cell refinement was carried out with “Crysalis”.[5] Face-indexed absorption corrections were applied using spherical harmonics, implemented in SCALE3 ABSPACK scaling algorithm. Using Olex2,[6] the structure was solved with the ShelXT[7] structure solution program using Intrinsic Phasing and refined with the ShelXL[8] refinement package using Least Squares minimisation.

Crystals of equilibrium mixture of both **2** and **3**, and **2** and **6,** both revealed the presence of [Ir(amine)(η^2-CO_3)(IMes)(η^2-imine)] which has been previously reported [4] Here, a different crystal structure was observed which contained a DCM of crystallization rather than the phenthylamine/H_2O of crystallization in the previously reported unit cells.

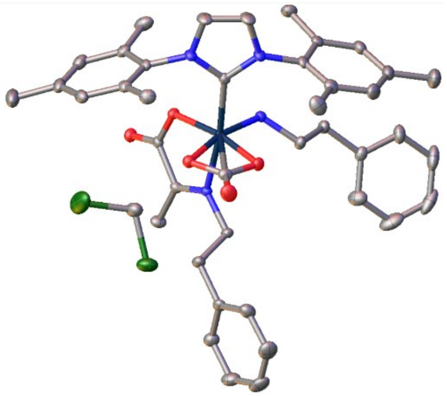

***Figure S7*) Crystal structure**

***Table S6*) Crystal data and structure refinement.**

Compound	**2 and 3**
Empirical formula	$C_{42}H_{49}Cl_2IrN_4O_5$
Formula weight	952.95
Crystal system	monoclinic
Space group	$P2_1/n$
a/Å	14.08570(10)
b/Å	11.99650(10)
c/Å	24.9643(2)
α/°	90
β/°	97.6750(10)
γ/°	90
Volume/Å^3	4180.65(6)
Z	4
ρ_{calc}g/cm^3	1.514
μ/mm^{-1}	3.369
F(000)	1920.0
Crystal size/mm^3	0.151 × 0.139 × 0.09
Radiation	MoKα (λ = 0.71073)
2Θ range for data collection/°	6.588 to 63.576
Index ranges	-20 ≤ h ≤ 20, -17 ≤ k ≤ 17, -36 ≤ l ≤ 36
Reflections collected	63675
Independent reflections	13379 [R_{int} = 0.0359, R_{sigma} = 0.0291]
Data/restraints/parameters	13379/0/502
Goodness-of-fit on F^2	1.043
Final R indexes [I>=2σ (I)]	R_1 = 0.0219, wR_2 = 0.0437
Final R indexes [all data]	R_1 = 0.0289, wR_2 = 0.0465
Largest diff. peak/hole / e Å^{-3}	0.93/-0.64

S4 Density Functional Theory (DFT) calculations

S4.1: General Remarks

All DFT calculations were performed on the full molecule (without simplification) using the Gaussian 09 software package.[9] All structures were optimized in combination with solvent effects modelled with the integral equation formalism variant of the Polarizable Continuum Model (IEFPCM).[10-12] All calculations had the solvent specified as dichloromethane. All calculations used the PBE0 DFT functional[13] and the basis set family defined as def2-SVP from Ahlrichs[14, 15] for all atoms (taken from the EMSL website[16, 17]) except hydride atoms and iridium. The hydride atoms were assigned the larger def2-TZVPP basis set[14, 15] and iridium was assigned the LANL08(f) basis set with the associated effective core potential (ECP).[18] Frequency calculations were used to confirm that the structures obtained were local minima along with zero-point energies and thermal corrections to the energy at 298.15 K. Single point calculations (again with solvation) were then undertaken with all atoms apart from Iridium assigned the larger basis sets from the def2-TZVPP family (the LANL08(f) basis set was maintained for iridium). These calculations also included the GD3BJ empirical dispersion correction from Grimme which includes Beck-Johnson dampling.[19] The thermal energy corrections were then applied to obtain chemical enthalpies and free energies.[20] This approach has previously been used to model the reactions of similar systems.[21] The calculations were checked for Basis Set Superposition Errors (BSSE). The resulting counterpoise calculation[22, 23] revealed that errors of around 5 kJ mol^{-1} were present in all systems and so corrections were applied appropriately.

S4.2: Optimized geometry of the 5 coordinate intermediate

a)

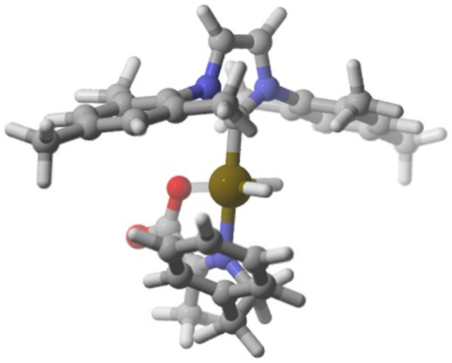

b)

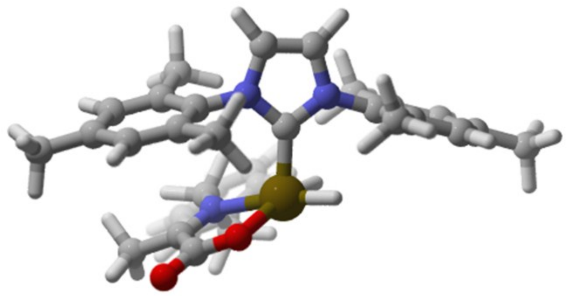

Figure S8) DFT optimised structure of the 5 coordinate intermediate resulting from ligand loss from a) 2A and b) 2B.

S4.3: DFT Results

Table S7) Relative energies of 1 and thermodynamic parameters related to hydrogen exchange processes at 298 K. All values are in kJ mol^{-1} and relative to 1A.

Complex	ΔH	ΔG	ΔH H_2 loss	ΔG H_2 loss	ΔH L loss	ΔG L loss	ΔH H_2 addition to 5 coordinate intermediate	ΔG H_2 addition to 5 coordinate intermediate
**1A**	0	0	136.7	104.6	94.7	41.1	32.8	11.4
**1B**	− 4.0	− 0.9	126.2	99.9	141.4	92.7	53.1	35.7
**1C**	9.5	20.3	126.2	99.9	141.4	92.7	53.1	35.7

Table S8) Relative energies at 298 K predicted from DFT calculations. All values are in kJ mol^{-1} and relative to 2A. 2A-Acetonitrile and 2A-thiophene refers to theoretical complexes in which phenethylamine is replaced by acetonitrile or thiophene respectively. The structures of these complexes is given in Scheme 1 of the main paper.

Complex	ΔH	ΔG	ΔH L loss	ΔG L loss
**2A**	0	0	84.4	33.6
**2B**	− 10.5	− 2.6	117.4	62.7
**3A**	− 7.4	− 8.3	-	-
**3B**	5.3	12.7	-	-
**4A**	− 6.1	− 4.5	-	-
**4B**	− 1.9	− 0.6	-	-
**5A**	− 9.6	− 0.4	-	-
**5B**	− 15.7	3.9	-	-
**7A**	41.8	34.0	-	-
**2A-Thiophene**	23.8	18.6	-	-
**2A-Acetonitrile**	21.2	12.5	-	-

S5 Kinetic modelling and rate constants

S5.1: Kinetic modelling of transmission rates of formation of 3-5 from 2.

A series of ^{1}H NMR spectra were recorded after the addition of coligand L to **2**. The proportion of each of the four complexes: **2A**, **2B**, **LA** and **LB** (where L=3, 4, 5 for pyridine, imidiazole and DMSO respectively) was determined on the basis of the integral intensities of the hydride resonance *trans* to nitrogen which are free from spectral overlap. This time course data was fit to a kinetic model allowing for the exchange of all four species with the 12 rate constants described in Figure S5.1a and Equations 3-8 where $[X]_{t-\delta t}$ and $[X]_t$ are the concentration or proportion of species **X** in solution at t=t-δt and t=t where ∂t is the incremental time difference, Am is Amine, and L is the added coligand (L=3=pyridine, L=4=imidiazole, L=5=DMSO). Rate constants were found by minimizing the sum of the squared differences between experimental and predicted values. Values of $[2A]_0$, $[2B]_0$,$[LA]_0$, $[LB]_0$ were taken from ^{1}H NMR integral intensities whereas those of $[L]_0$ and $[Am]_0$ were allowed to change. It was essential to exclude some initial data at short reaction times due to the release of large amounts of free amine upon its replacement by coligand in **2A.** These large initial fluctuations in $[Am]_0$ have an effect on the equilibrium between amine and imine in solution.

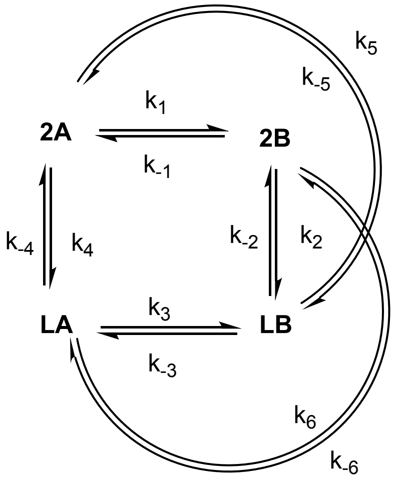

Figure S9) Exchange model used for kinetic modelling

$$[2A]_t = [2A]_{t-\delta t} + \left(-k_1[2A] + k_{-1}[2B] - k_5[L][2A] + k_{-5}[LB][Am] - k_4[2A][L] + k_{-4}[LA][Am]\right)\partial t \quad (3)$$

$$[2B]_t = [2B]_{t-\delta t} + \left(k_1[2A] - k_{-1}[2B] - k_2[L][2B] + k_{-2}[LB][Am] - k_{-6}[2B][L] + k_6[LA][Am]\right)\partial t \quad (4)$$

$$[LA]_t = [LA]_{t-\delta t} + \left(-k_{-4}[LA][Am] + k_4[2A][L] - k_3[LA] + k_{-3}[LB] - k_6[LA][Am] + k_{-6}[2B][L]\right)\partial t \quad (5)$$

$$[LB]_t = [LB]_{t-\delta t} + \left(k_2[2B][L] - k_{-2}[LB][Am] - k_{-5}[LB][Am] + k_5[2A][L] - k_{-3}[LB] + k_3[LA]\right)\partial t \quad (6)$$

$$[L]_t = [L]_{t-\delta t} + (-k_4[2A][L] + k_{-4}[LA][Am] - k_2[2B][L] + k_{-2}[LB][Am] + k_6[LA][Am] - \text{[illegible]} \partial t \quad (7)$$

$$[Am]_t = [Am]_{t-\delta t} + (k_2[2B][L] - k_{-2}[LB][Am] - k_6[LA][Am] + k_{-6}[2B][L] + k_5[2A][L] + k \text{[illegible]} \partial t \quad (8)$$

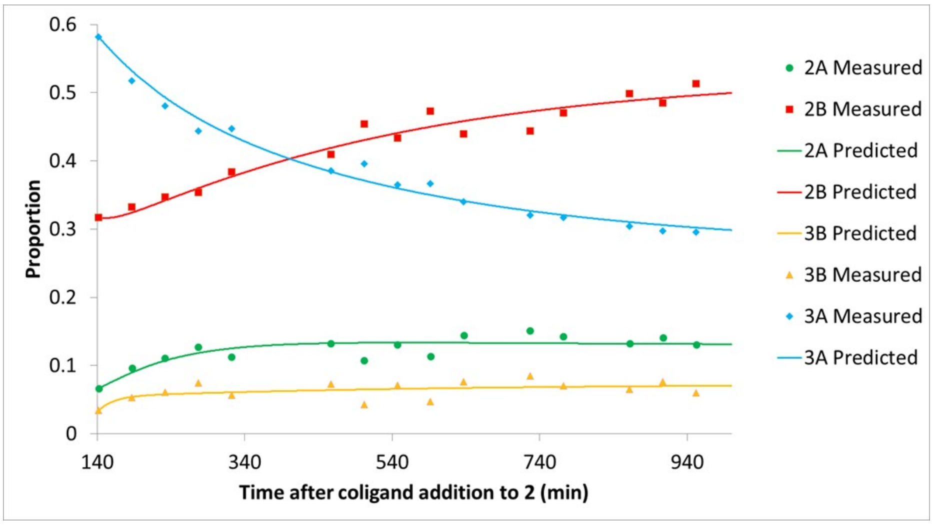

Figure S10) Kinetic modelling showing experimental data (markers) and predicted data (solid lines) after the addition of pyridine to 2. The transmission rates used to fit this data are shown in Table S9.

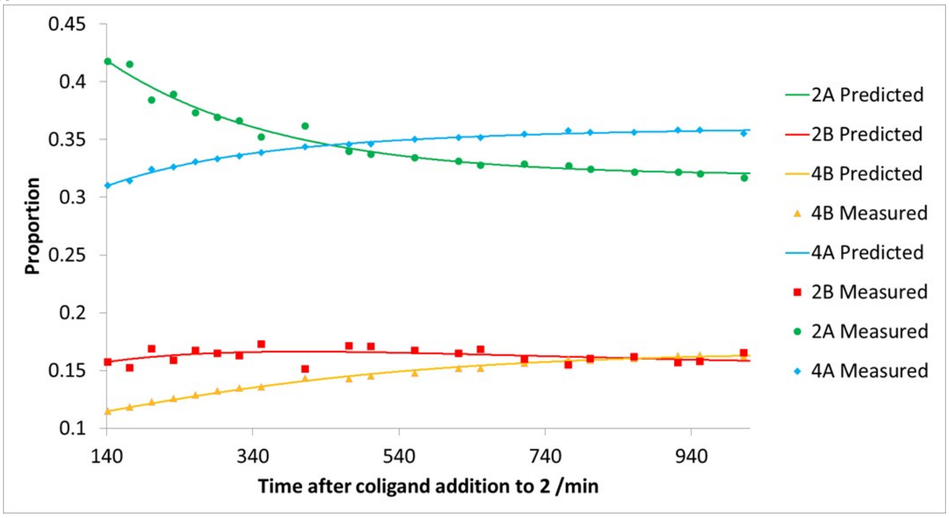

Figure S11) Kinetic modelling showing experimental data (markers) and predicted data (solid lines) after the addition of imidazole to 2. The transmission rates used to fit this data are shown in Table S9.

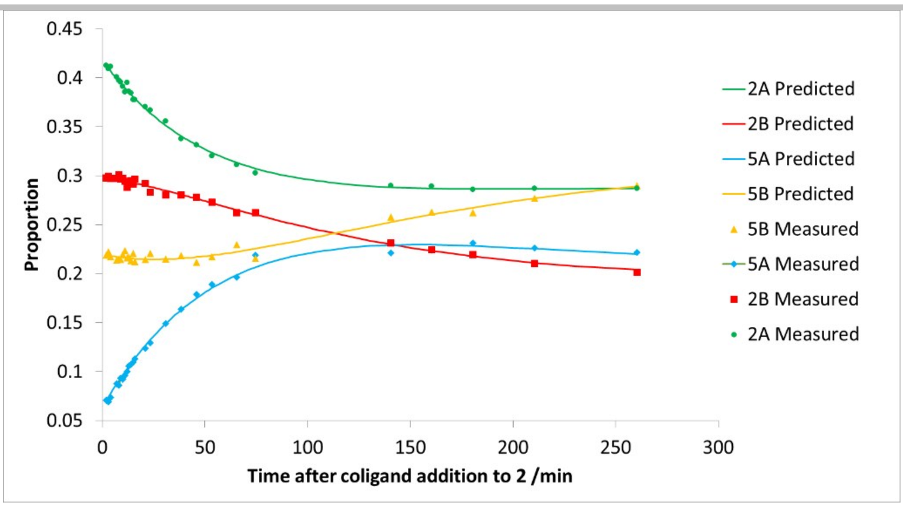

Figure S12) Kinetic modelling showing experimental data (markers) and predicted data (solid lines) after the addition of DMSO to 2. The transmission rates used to fit this data are shown in Table S9.

Table S9) Transmission rates used to fit kinetic data shown in Figures S10-12 according to the model shown in Figure S9 and Equations 3-8. Note that rate constants and errors less than 1 X 10^{-6} s^{-1} were set by this model to 0.

	Pyridine $/10^{-5}$ s^{-1}	Imidazole $/10^{-5}$ s^{-1}	DMSO $/10^{-5}$ s^{-1}
k_1	2.9 ± 2.5	1.7 ± 0.7	10.6 ± 1.2
k_2	0.7 ± 0.3	0.2[a]	0.0
k_3	2.7 ± 0.3	1.6 ± 2.3	9.9 ± 1.0
k_4	0.9 ± 0.2	0.3[a]	0.6 ± 0.1
k_5	0.2 ± 0.6	0.0	0.0
k_6	0.0	0.0	0.0
k_{-1}	0.0 ± 0.3	0.0	0.0
k_{-2}	4.9 ± 1.6	0.0	0.0
k_{-3}	0.0	5.1 ± 2.3	0.0
k_{-4}	0.2[a]	0.4[a]	1.3 ± 0.1
k_{-5}	2.0 ± 0.1	0.0	0.6 ± 0.1
k_{-6}	0.0	0.2 ± 0.3	1.7 ± 0.1

[a] According to the model these numbers are well defined with errors substantially less than the quoted value

We note that these are observed transmission rate constants. In the main paper we refer to k_4 as $k_{trans\mathbf{2ALA}}$ and k_2 as $k_{trans\mathbf{2BLB}}$. These data are consistent with faster rates of coligand replacement in **2A** compared to **2B** (as the transmission rate constant k_4 is greater than k_2). This data is also consistent with pyridine binding faster than imidazole (as transmission rate constant k_4 and k_2 are higher).

S5.2: Kinetic modelling and transmission rates of formation of 3 and 4 from 2

A series of ^{1}H NMR spectra were recorded after the addition of an equimolar solution of pyridine and imidiazole in 20 μL DCM-d_2 to an equilibrium mixture of **2**. The proportion of each of the six complexes: **2A**, **2B**, **3A, 3B**, **4A** and **4B** were determined from the integral intensities of the hydride resonance *trans* to nitrogen which are free from spectral overlap. This time course data was fit to a kinetic model allowing for the exchange of all six species with the 30 rate constants described in Figure S5.2a and Equations 9-17 where $[X]_{t-\delta t}$ and $[X]_t$ are the concentration or proportion of species **X** in solution at t= $t-\delta t$ and t=t where ∂t is the incremental time difference, Am is Amine, Py is pyridine and Im is imidiazole. Rate constants were found by minimizing the sum of the squared differences between experimental and predicted values. Values of $[2-4A]_0$ and $[2-4B]_0$ were taken from ^{1}H NMR integral intensities whereas those of $[Am]_0$, $[Py]_0$ and $[Im]_0$ were allowed to change. Some initial data points immediatley after coligand addition were omitted due to non equilibirum behaviour including large initial fluctuations in $[Am]_0$ having an effect on the equilibrium between amine and imine in solution.

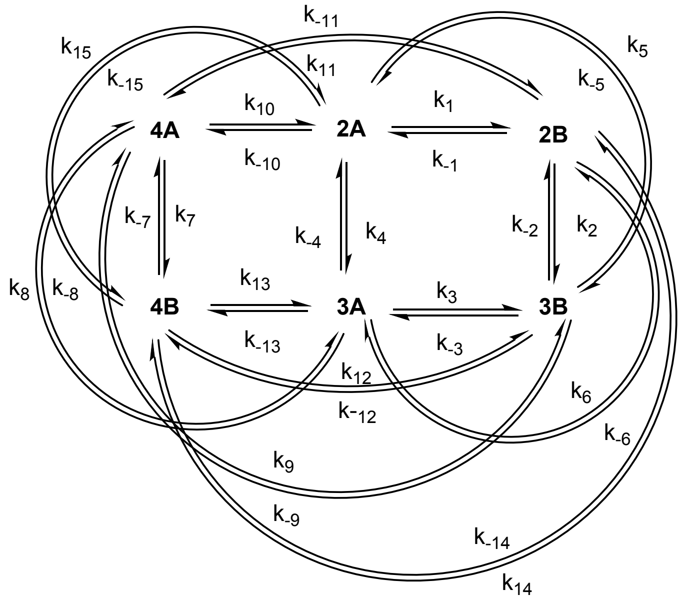

Figure S13) Exchange model used for kinetic modelling

$$[2A]_t = [2A]_{t-\delta t} + \big(-k_1[2A] + k_{-1}[2B] - k_5[L][2A] + k_{-5}[3B][Am] - k_4[2A][Py] + k_{-4}[3\ldots$$
∂t (9)

$$[2B]_t = [2B]_{t-\delta t} + \big(k_1[2A] - k_{-1}[2B] - k_2[Py][2B] + k_{-2}[3B][Am] - k_{-6}[2B][Py] + k_6[3A]\ldots$$
∂t (10)

$$[3A]_t = [3A]_{t-\delta t} + \big(-k_{-4}[3A][Am] + k_4[2A][Py] - k_3[3A] + k_{-3}[3B] - k_6[3A][Am] + k_{-6}\ldots$$
∂t (11)

$$[3B]_t = [3B]_{t-\delta t} + \big(k_2[2B][Py] - k_{-2}[3B][Am] - k_{-5}[3B][Am] + k_5[2A][Py] - k_{-3}[3B] + k\ldots$$
∂t (12)

$$[4A]_t = [4A]_{t-\delta t} + \big(k_8[3A][Im] - k_{-8}[4A][Py] - k_9[4A][Py] + k_{-9}[3B][Im] - k_7[4A] + k_{-7}\ldots$$
∂t (13)

$$[4B]_t = [4B]_{t-\delta t} + \big(-k_{12}[4B][Py] + k_{-12}[3B][Im] + k_{-13}[3A][Im] - k_{13}[4B][Py] - k_{-7}[4\ldots$$
∂t (14)

$$[Am]_t = [Am]_{t-\delta t} + \big(k_4[2A][Py] - k_{-4}[3A][Am] + k_2[2B][Py] - k_{-2}[3B][Am] - k_6[3A][Am]\ldots$$
∂t (15)

$$[Py]_t = [Py]_{t-\delta t} + \big(-k_4[2A][Py] + k_{-4}[3A][Am] - k_2[2B][Py] + k_{-2}[3B][Am] + k_6[3A][A\ldots$$
∂t (16)

$$[Im]_t = [Im]_{t-\delta t} + \big(-k_8[3A][Im] + k_{-8}[4A][Py] - k_{-9}[3B][Im] + k_9[4A][Py] - k_{12}[3B][Im\ldots$$
∂t (17)

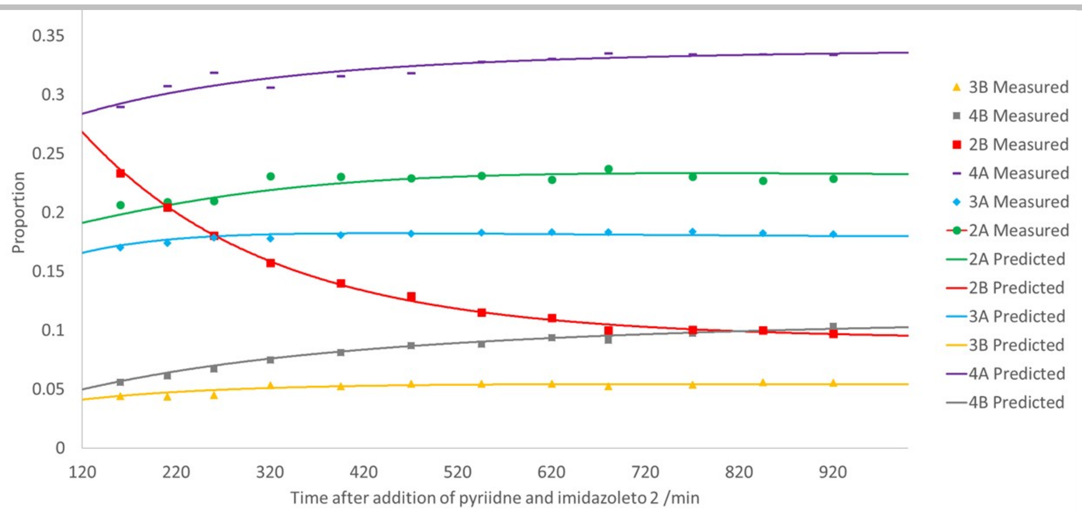

Figure S14) Kinetic modelling showing experimental data (markers) and predicted data (solid lines) after the addition of an equimolar solution of pyridine and imidazole to 2. The rate constants used to fit this data are shown in Table S10

Table S10) Transmission rates used to fit kinetic data shown in Figure S14 according to the model shown in Figure S13 and Equations 9-17. Here, all errors were less than the final significant figure quoted.

	$/10^{-5}$ s^{-1}		$/10^{-5}$ s^{-1}
k_1	0.22	k_{-1}	0.07
k_2	0.06	k_{-2}	0.22
k_3	0.15	k_{-3}	0.001
k_4	0.48	k_{-4}	0.23
k_5	0.18	k_{-5}	0.11
k_6	0.13	k_{-6}	0.002
k_7	0.83	$k\text{-}_7$	0.16
k_8	0.26	$k\text{-}_8$	0.001
k_9	0.18	$k\text{-}_9$	0.22
k_{10}	0.78	$k\text{-}_{10}$	0.10
k_{11}	0.22	$k\text{-}_{11}$	0.09
k_{12}	0.30	$k\text{-}_{12}$	0.23
k_{13}	0.13	$k\text{-}_{13}$	0.002
k_{14}	0.001	$k\text{-}_{14}$	0.002
k_{15}	0.002	$k\text{-}_{15}$	0.002

We note that these are observed transmission rate constants. These data are consistent with faster rates of coligand replacement in **2A** compared to **2B**. This is true for amine replacement with pyridine ($k_4 > k_2$), amine replacement with imidazole ($k_{-10} > k_{14}$), pyridine replacement with imidazole ($k_8 > k_{-12}$), and the reverse replacements (transmission rates $k_{-4} > k_{-2}$, $k_{-10} > k_{-14}$ although $k_{-8} < k_{12}$ respectively). We note that pyridine binds faster than imidazole as transmission rates $k_2 > k_{14}$ and $k_4 > k_{-10}$.

S6: Hydrogen exchange rates

The exchange rate of the hydride ligands of these complexes with free hydrogen was determined using exchange spectroscopy (EXSY). In these measurements the hydride signal *cis* to oxygen was selectively excited and after set time delay this resonance decreases in intensity and evolves into resonances for free hydrogen (δ 4.6), and the inequivalent hydride ligand, H_b. The integrals from the NMR spectra at various time delays are interpreted as percentage abundance of bound and free hydrogen. A three site exchange model is used to fit exchange rates as previously reported.[4]

S7: ^{15}N Hyperpolarisation studies

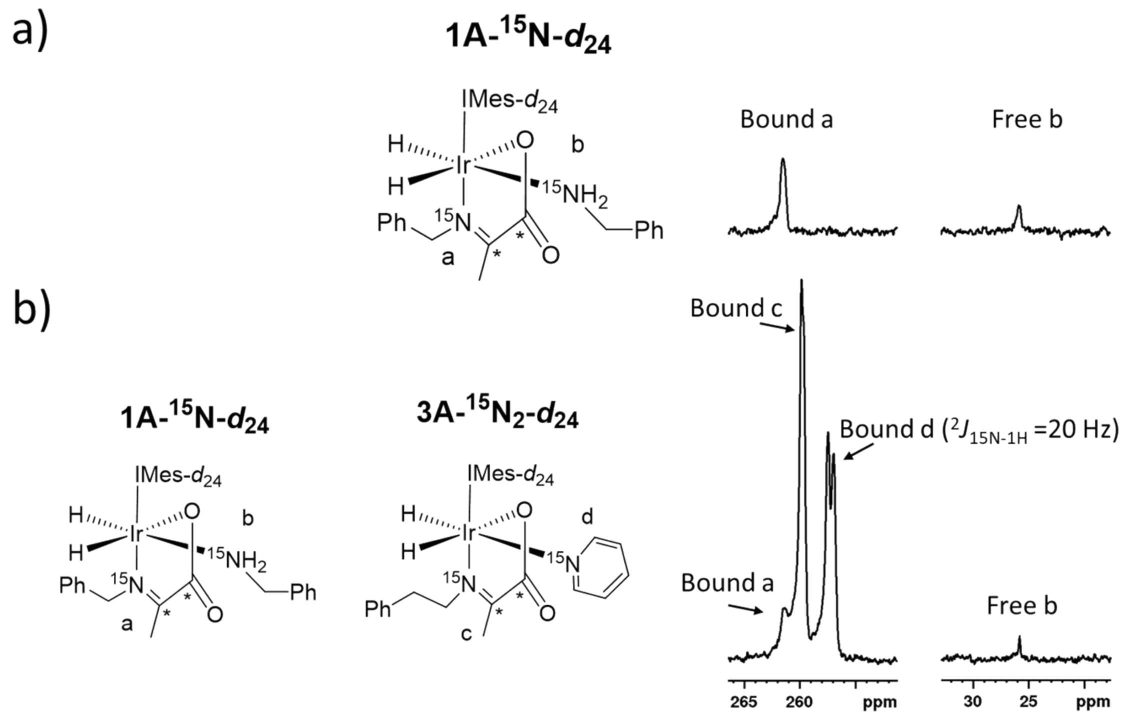

Figure S15) Partial hyperpolarized ^{15}N NMR spectra of a) 1A-^{15}N-d_{24} and b) an equilibrium mixture of 1A-^{15}N-d_{24} and 3A-$^{15}N_2$-d_{24} recorded after 10 seconds shaking in a mu metal shield

S8: Hyperpolarized singlet decay measurements

Samples were polarized by shaking in the stray field of the magnet (65 G) for 10 seconds. Decay profiles were obtained by leaving the hyperpolarized samples inside a mu metal shield for different time periods and measuring the ^{13}C signal intensity that results. Decay profiles were recorded at differing *p*-H_2 pressures and were fitted to a biexponential decay as previously reported[4]

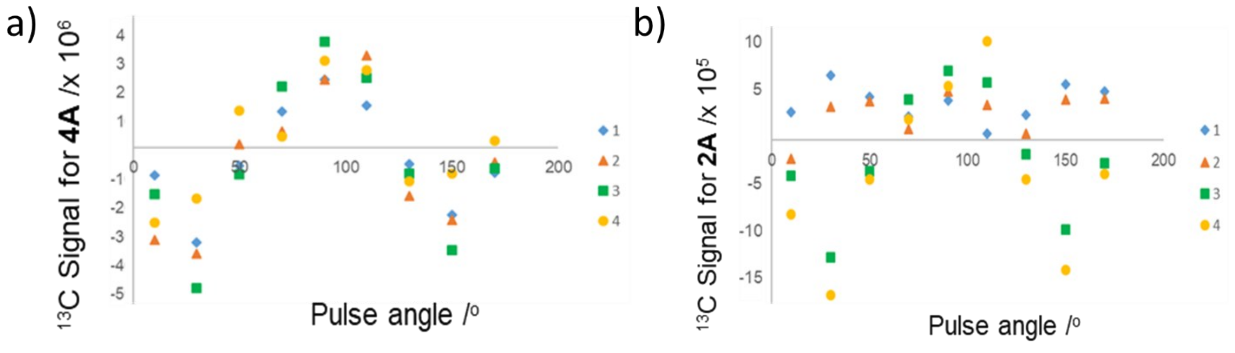

Figure S16) The hyperpolarized profile for an equilibrium mixture of 4A (a) and 2A varies (b) as a function of observation pulse angle. Integrated hyperpolarized resonances for 4A and 2A as a function of pulse angle are shown with the lines 1-4 corresponding to the coupled resonances of the $^{13}C_2$ as assigned from downfield to upfield.

References

1. R. W. Adams, J. A. Aguilar, K. D. Atkinson, M. J. Cowley, P. I. Elliott, S. B. Duckett, G. G. Green, I. G. Khazal, J. López-Serrano and D. C. Williamson, *Science*, 2009, **323**, 1708-1711.
2. L. D. Vazquez-Serrano, B. T. Owens and J. M. Buriak, *Inorg. Chim. Acta*, 2006, **359**, 2786-2797.
3. W. Iali, P. J. Rayner, A. Alshehri, A. J. Holmes, A. J. Ruddlesden and S. B. Duckett, *Chem. Sci.* , 2018, **9**, 3677-3684.
4. B. J. Tickner, W. Iali, S. S. Roy, A. C. Whitwood and S. B. Duckett, *ChemPhysChem*, 2019, **20**, 241-245.
5. O. D. Ltd., *Journal*, Version 1.171.34.41.
6. O. V. Dolomanov, L. J. Bourhis, R. J. Gildea, J. A. Howard and H. Puschmann, *J. Appl. Cryst.*, 2009, **42**, 339-341.
7. G. Sheldrick, *Acta Crys. Sec. A.*, 2015, **71**, 3-8.
8. G. Sheldrick, *Acta Crys. Sec. C.*, 2015, **71**, 3-8.
9. G. W. T. M. J. Frisch, H. B. Schlegel, G. E. Scuseria, , J. R. C. M. A. Robb, G. Scalmani, V. Barone, B. Mennucci, , H. N. G. A. Petersson, M. Caricato, X. Li, H. P. Hratchian, , J. B. A. F. Izmaylov, G. Zheng, J. L. Sonnenberg, M. Hada, , K. T. M. Ehara, R. Fukuda, J. Hasegawa, M. Ishida, T. Nakajima, , O. K. Y. Honda, H. Nakai, T. Vreven, J. A. Montgomery, Jr., , F. O. J. E. Peralta, M. Bearpark, J. J. Heyd, E. Brothers, , V. N. S. K. N. Kudin, T. Keith, R. Kobayashi, J. Normand, , A. R. K. Raghavachari, J. C. Burant, S. S. Iyengar, J. Tomasi, , N. R. M. Cossi, J. M. Millam, M. Klene, J. E. Knox, J. B. Cross, , C. A. V. Bakken, J. Jaramillo, R. Gomperts, R. E. Stratmann, , A. J. A. O. Yazyev, R. Cammi, C. Pomelli, J. W. Ochterski, , K. M. R. L. Martin, V. G. Zakrzewski, G. A. Voth, , J. J. D. P. Salvador, S. Dapprich, A. D. Daniels, , J. B. F. O. Farkas, J. V. Ortiz, J. Cioslowski, and G. and D. J. Fox, Inc., Wallingford CT, 2013, *Journal*, 2010.
10. E. Cances, B. Mennucci and J. Tomasi, *Journal of Chemical Physics*, 1997, **107**, 3032-3041.
11. B. Mennucci, E. Cances and J. Tomasi, *Journal of Physical Chemistry B*, 1997, **101**, 10506-10517.
12. E. Cances and B. Mennucci, *Journal of Mathematical Chemistry*, 1998, **23**, 309-326.
13. C. Adamo and V. Barone, *J. Chem Phys.*, 1999, **110**, 6158-6170.
14. F. Weigend and R. Ahlrichs, *Physical Chemistry Chemical Physics*, 2005, **7**, 3297-3305.
15. A. Schafer, H. Horn and R. Ahlrichs, *Journal of Chemical Physics*, 1992, **97**, 2571-2577.
16. D. Feller, *Journal of Computational Chemistry*, 1996, **17**, 1571-1586.
17. K. L. Schuchardt, B. T. Didier, T. Elsethagen, L. S. Sun, V. Gurumoorthi, J. Chase, J. Li and T. L. Windus, *Journal of Chemical Information and Modeling*, 2007, **47**, 1045-1052.
18. L. E. Roy, P. J. Hay and R. L. Martin, *Journal of Chemical Theory and Computation*, 2008, **4**, 1029-1031.
19. S. Grimme, S. Ehrlich and L. Goerigk, *J. Comput. Chem.*, 2011, **32**, 1456-1465.
20. C. R. M. Buehl, D. Pantazis, T. Bredow and F. Neese, *J. Chem. Theor. Comput.*, 2008, **4**, 1449-1459.
21. J. E. Richards, A. J. J. Hooper, O. W. Bayfield, M. C. R. Cockett, G. J. Dear, A. J. Holmes, R. O. John, R. E. Mewis, N. Pridmore, A. D. Roberts, A. C. Whitwood and S. B. Duckett, *Chemical Communications*, 2018, **54**, 10375-10378.
22. S. F. Boys and F. Bernadi, *Molecular Physics*, 1970, **19**, 553-553.
23. S. Simon, M. Duran and J. J. Dannenberg, *Journal of Chemical Physics*, 1996, **105**, 11024-11031.

Author Contributions

Complexes were synthesized and hyperpolarized by B.J.T. and NMR characterization was performed by B.J.T. All DFT calculations were performed by R. O. J. Hydrogen exchange rates were measured by B.J.T. Singlet state measurements were recorded by B.J.T. and S.S.R. X-ray diffraction data was collected and analyzed by S. J. H and A. C. W. Results were discussed by all who contributed to the paper which was written by B.J.T. and S.B.D.
